# Transcriptomic insights into the effects of sublethal exposure to endolysin LNT113 on *Escherichia coli*

**DOI:** 10.71150/jm.2605009

**Published:** 2026-07-31

**Authors:** Heymin Song, Nanjoo Park, Eunsuk Kim, Seowon Jang, Sunghoon Kim, Jeongik Cho, Hyunjin Yoon

**Affiliations:** 1 Department of Molecular Science and Technology, Ajou University, Suwon 16499, Republic of Korea; 2 Division of Food Technology and Nutrition, Sun Moon University, Asan 31460, Republic of Korea; 3 College of Advanced Bio-Convergence Engineering, Ajou University, Suwon 16499, Republic of Korea

**Keywords:** engineered endolysin, LNT113, transcriptome, *Escherichia coli*

## Abstract

The global rise of multidrug-resistant bacteria poses a critical threat to public health, and bacteriophage-derived endolysins have emerged as promising alternatives to conventional antibiotics. The engineered endolysin LNT113, derived from the *Escherichia coli* phage PBEC131 endolysin EC340, exhibits potent lytic activity against Gram-negative bacteria. This study investigated the transcriptomic responses of *E. coli* to sublethal LNT113 stress and identified genetic determinants required for bacterial adaptation to endolysin-induced stress. Transcriptomic analysis identified 552 differentially expressed genes (DEGs) following sublethal LNT113 exposure. Thirteen DEGs associated with stress response and envelope maintenance were individually deleted to generate thirteen mutant strains and to functionally evaluate their roles in bacterial stress tolerance. Among these, the Δ*fabB* and Δ(*prmB*–*yfcL*) mutants exhibited significantly reduced survival under sublethal LNT113 exposure, indicating increased susceptibility to the endolysin. Regarding the *prmB*–*yfcL* operon, individual genes were deleted to determine the gene critical for bacterial tolerance. Deletion of *aroC* and *mepA* rendered *E. coli* more susceptible to LNT113. Furthermore, 1-N-phenylnaphthylamine uptake assays demonstrated increased membrane permeability in the Δ*fabB*, Δ*aroC*, and Δ*mepA* mutants. Complementation with pWSK129::*fabB*, pWSK129::*aroC*, and pWSK129::*mepA* restored membrane integrity in the respective mutant strains. These findings suggest that *fabB*-mediated unsaturated fatty acid biosynthesis and *mepA*-dependent peptidoglycan remodeling are critical for maintaining envelope integrity under endolysin stress, whereas *aroC* may indirectly support bacterial tolerance to LNT113 via metabolic adaptation. This study provides insights into bacterial responses to LNT113 and offers a foundation for optimizing endolysin-based therapeutic strategies.

## Introduction

The increasing prevalence of antimicrobial-resistant infections represents a critical challenge to global health (Naghavi et al., 2024). In particular, antibiotic resistance poses a major obstacle in the treatment of infections caused by Gram-negative bacteria (WHO, 2017). The development of new antibiotics or alternative antibacterial strategies is essential to combat multidrug-resistant (MDR) bacteria and mitigate antimicrobial resistance. Bacteriophage-derived endolysins are lytic enzymes that induce cell lysis by degrading the peptidoglycan layer of bacterial cell walls at the end of the lytic replication cycle (Grabowski et al., 2021). Their enzymatic activities vary according to their mechanisms of peptidoglycan cleavage and include glucosaminidases, muramidases, lytic transglycosylases, endopeptidases, and amidases (Abdelrahman et al., 2021). Because endolysins target peptidoglycan structures conserved exclusively in bacteria, they represent promising antibacterial agents against MDR Gram-negative bacteria (Sisson et al., 2024).

During the final stage of the reproductive cycle, bacteriophages encode membrane proteins, such as holins and spanins, to facilitate endolysin transport across the cytoplasmic membrane, enabling access to and degradation of the peptidoglycan layer (Young, 2013). However, when used as alternatives to conventional antibiotics, endolysins are applied exogenously. In Gram-positive bacteria, endolysins can directly access and degrade the exposed peptidoglycan layer of the cell wall. In contrast, Gram-negative bacteria possess an outer membrane that must be traversed before endolysins can access the peptidoglycan layer. Accordingly, the low permeability of the outer membrane limits the exogenous application of endolysins against Gram-negative bacteria, and some endolysins require additional strategies to overcome this barrier (Aitken et al., 2025).

Fusion of a cell membrane–permeabilizing peptide to Gram-negative endolysins has been widely employed to enhance antibacterial efficacy (Aitken et al., 2025). LNT113 is an engineered endolysin derived from endolysin EC340 of *Escherichia coli* bacteriophage PBEC131(Hong et al., 2022). In a previous study, antibacterial activity was enhanced via targeted amino acid substitutions within the enzymatic activity domain (mtEC340) and N-terminal fusion with the antimicrobial peptide cecropin A (LNT113), resulting in superior bactericidal activity at low concentrations (Cho et al., 2024; Hong et al., 2022). This enhanced lytic activity may improve therapeutic feasibility, reduce production costs, and facilitate large-scale manufacturing, thereby addressing key barriers to the practical use of endolysins in healthcare settings (Khan et al., 2024).

Regarding concerns about the development of bacterial resistance to endolysins, no resistant mutants were detected in bacterial populations repeatedly exposed to endolysins or artilysins at subinhibitory doses (Briers et al., 2014; Fischetti, 2005). However, despite the low likelihood of resistance development to endolysins and artilysins relative to conventional antibiotics, emerging evidence indicates that sublethal exposure to endolysins may lead to unintended consequences. A previous study showed that exposure to subinhibitory concentrations of the engineered endolysin LysPA90 resulted in increased bacterial motility and upregulation of the expressions of virulence-associated genes in adherent-invasive *E. coli* (Hwang et al., 2022). These findings suggest that sublethal endolysin exposure may induce bacterial stress responses or adaptive mechanisms, potentially influencing bacterial physiology and posing challenges to their therapeutic application.

This study aimed to systematically analyze bacterial molecular responses to sublethal concentrations of LNT113 using transcriptomic analysis. In particular, we focused on the effects of LNT113 on the expression of genetic determinants involved in tolerance to and recovery from endolysin-mediated damage. The findings of this study provide a scientific basis for establishing optimal usage strategies for LNT113, thereby maximizing its potential as an alternative to conventional antibiotics in clinical applications.

## Materials and Methods

### Bacterial strains and growth conditions

Details of the bacterial strains used in this study are provided in Table S1. Bacterial strains were cultured in Luria–Bertani (LB) broth at 37°C. Deletion mutants were constructed using the λ Red recombination system (Datsenko and Wanner, 2000), with *Escherichia coli* K-12 MG1655 (ATCC 700926) as the parental strain. Briefly, the kanamycin resistance cassette was amplified using polymerase chain reaction (PCR) from plasmid pKD13 using primers containing 40 bp sequences homologous to the flanking regions of the target gene. The PCR products were introduced into *E. coli* harboring pKD46, and kanamycin-resistant recombinants were subsequently confirmed via PCR using diagnostic primers. To remove the resistance cassette, cells were transformed with plasmid pCP20 encoding the flippase recombinase, and markerless deletions were confirmed using diagnostic PCR. All primers used in this study are listed in Table S2.

### Construction of recombinant plasmids

The *fabB*, *aroC*, and *mepA* genes were amplified using the primers listed in Table S3 and cloned into the low-copy plasmid pWSK129 (Wang and Kushner, 1991) via the ApaI and XbaI restriction sites. For *aroC* and *mepA* expression, the putative transcriptional regulatory region (500 bp upstream of *prmB*) was amplified via PCR using promoter CF and promoter CR primers and first inserted into pWSK129 through the EcoRI site. Subsequently, the coding sequences of *aroC* and *mepA* were cloned downstream of their native promoter region. All recombinant plasmids were verified by sequencing (Macrogen, Korea). The resulting constructs were then introduced into the corresponding mutant strains (Δ*fabB*, Δ*aroC*, and Δ*mepA*).

### Expression and purification of endolysin LNT113

The *E. coli* BL21(DE3) Star strain harboring the LNT113 expression plasmid was used for protein overexpression, as previously described (Hong et al., 2022). Protein expression was induced with 0.05 mM isopropyl β-D-1-thiogalactopyranoside during the logarithmic growth phase (OD_600_ = 0.6–0.8), and cultures were incubated at 25°C for 4 h. Harvested cells were resuspended in lysis buffer (20 mM Tris–HCl, pH 7.5, 300 mM NaCl, 20 mM imidazole) and disrupted by sonication. Cell debris was removed by centrifugation at 10,000 × g for 20 min at 4°C, and the clarified supernatant was filtered through a 0.2 μm membrane. The recombinant protein was purified using Ni–NTA His-tag affinity chromatography and subsequently dialyzed against storage buffer (20 mM Tris–HCl; pH 7.5, 150 mM NaCl). Purified LNT113 was analyzed by SDS–PAGE (Fig. S1), and protein concentration was measured using the Bradford assay (Bio-Rad, USA).

### RNA extraction

*E. coli* MG1655 cells were grown to the mid-logarithmic phase and collected by centrifugation. The harvested cells were washed with 20 mM Tris–HCl (pH 7.5) prior to treatment. For the Tris 0 min group, 4 × 10^9^ CFU were suspended in 20 mM Tris–HCl, and 5 × 10^8^ CFU were immediately harvested and stabilized using RNAprotect Bacteria Reagent (Qiagen, Germany). For the Tris 15 min group, cells resuspended in 20 mM Tris–HCl were incubated on ice for 15 min before RNA stabilization. For the LNT113 15 min group, 4 × 10^9^ CFU were treated with 0.64 μM LNT113 in 20 mM Tris–HCl and incubated on ice for 15 min, followed by RNA stabilization with RNAprotect Bacteria Reagent. Bacterial cells were centrifuged at 5,000 × g for 10 min, and total RNA was extracted using the RNeasy Mini Kit (Qiagen, Germany) according to the manufacturer’s instructions. All experiments were performed in biological triplicates.

### RNA sequencing and data analysis

Total RNA was subjected to ribosomal RNA depletion using the TruSeq Standard Total RNA Library Prep Kit with Ribo-Zero (Illumina, USA). Sequencing was performed in paired-end mode (2 × 150 bp) by Sanigen (Korea). Raw reads were quality-trimmed, mapped to the *E. coli* K-12 MG1655 reference genome (GenBank accession no. U00096), and quantified using CLC Genomics Workbench (Qiagen, Germany).

RNA sequencing generated 30,897,139 and 30,341,677 reads for the Tris buffer 0 min replicates; 26,141,946 and 24,974,198 reads for the Tris buffer 15 min replicates; 23,480,653 and 27,526,219 reads for the LNT113 15 min replicates. Alignment to the *E. coli* MG1655 reference genome resulted in mapping rates of at least 89% across all samples, indicating high-quality sequencing data. Principal component analysis showed tight clustering of biological replicates and clear separation of the Tris buffer 15 min and LNT113 15 min samples from the Tris buffer 0 min control (Fig. S2), indicating high reproducibility and treatment-dependent transcriptional differences. Transcript abundance was normalized to transcripts per million (TPM), and reproducibility among biological replicates was evaluated by calculating the coefficient of variation. Differentially expressed genes (DEGs) were defined as those with a log_2_ fold change ≥ 1 or ≤ –1 and an adjusted *p*-value < 0.05 (false discovery rate), corrected using the Benjamini–Hochberg method. Fold change values were calculated as TPM ratios between LNT113-treated and Tris buffer control samples at 15 min (i.e., TPM_LNT113 15 min treated_ / TPM_Tris 15 min treated_). Functional classification of DEGs was performed using the Kyoto Encyclopedia of Genes and Genomes (KEGG; https://www.genome.jp/kegg) and Clusters of Orthologous Groups (COG; https://www.ncbi.nlm.nih.gov/research/cog) databases. Heatmaps were generated using Gitools v2.3.1. All RNA-seq data were deposited in the NCBI Gene Expression Omnibus database under accession number GSE327881.

### Quantitative reverse transcription polymerase chain reaction (RT-qPCR)

Complementary DNA was synthesized from total RNA using the EcoDry^TM^ Premix with random hexamers (Takara, Japan) according to the manufacturer’s instructions. RT-qPCR was performed on a StepOnePlus^TM^ Real-Time PCR System (Applied Biosystems, USA) using THUNDERBIRD^TM^ SYBR^®^ qPCR Mix (TOYOBO, Japan). The housekeeping gene *rpoD* was used as an internal reference for normalization (Jiao et al., 2024). Ct values for each gene were normalized to *rpoD*, and relative expression levels were presented as ΔCt values. RT-qPCR was conducted at least three times using RNA samples from separately prepared cultures and ΔCt or ΔΔCt values were presented as the Mean ± standard deviation. Student’s t-test was applied to determine statistical significance (*p* < 0.05). Primer used in this study are listed in Table S4.

### Bacterial turbidity reduction assay

*E. coli* cells in the mid-logarithmic phase were harvested and resuspended in 20 mM Tris–HCl buffer (pH 7.5) to an OD_600_ of 1.0. The bacterial suspension was then treated with LNT113 and incubated at 37°C. Optical density at 600 nm was measured at 5-min intervals for 1 h using a Synergy HTX Microplate Reader (BioTek, USA) to monitor turbidity reduction. Each experiment was performed in triplicate. To investigate concentration-dependent lytic activity, cells were exposed to a series of LNT113 concentrations (0, 0.02, 0.04, 0.08, 0.16, 0.32, 0.64, and 1.28 μM), and bacterial lysis was evaluated by measuring OD_600_ reduction over time. For quantitative analysis of mutant susceptibility, cells were treated with 0.64 μM LNT113. The change in optical density (Δ OD_600_) was calculated by subtracting the OD_600_ value at 0 min from the values obtained at 15, 30, and 60 min. Relative differences in turbidity reduction (ΔΔ OD_600_) were determined as Δ OD_600 [mutant]_ – Δ OD_600 [wild-type]_. All assays were conducted in triplicate.

### Bacterial viability test

*E. coli* cells (4 × 10^9^ cells) were resuspended in Tris–HCl buffer (pH 7.5) and treated with 0.64 μM LNT113 for 15 min. The bacterial cells were serially diluted and plated onto LB agar to enumerate live cells.

### 1-N-phenylnaphthylamine (NPN) uptake assay

Bacterial membrane permeability was assessed using the NPN uptake assay, as previously described with minor modifications (Helander and Mattila-Sandholm, 2000). *E. coli* MG1655 cells were grown to the mid-logarithmic phase (1 × 10^9^ CFU/ml). Harvested cells were resuspended in 20 mM Tris–HCl buffer (pH 7.5) and aliquoted into 96-well black plates (100 μl/well). LNT113 was added to a final concentration of 0.64 μM (50 μl), followed by the addition of 50 μl NPN (final concentration, 10 μM). Ethylenediaminetetraacetic acid (final concentration, 1 mM) was used as a positive control. After incubation at 37°C for 5 min, fluorescence was recorded using a Synergy HTX Microplate Reader (BioTek, USA) with excitation at 350 nm and emission at 420 nm.

### Statistical analysis

Statistical analyses were performed using GraphPad Prism 5 (GraphPad Software Inc., USA). All values are presented as the Mean ± standard deviation. Student’s t-test was mainly used to determine statistical significance. For comparisons involving more than two groups, one-way analysis of variance (ANOVA) followed by Tukey’s multiple-comparisons test was applied. Unless otherwise indicated, differences were considered not significant (n.s.). *p*-values are denoted as follows: *p < 0.05, **p < 0.01.

## Results

### Determination of a sublethal LNT113 concentration for *E. coli* RNA extraction

LNT113 is an engineered endolysin possessing a phage-derived muramidase domain and exhibits dose- and time-dependent antibacterial activity, resulting in 2-log and 4-log reductions in viable cells at concentrations of 0.3 µM and 0.9 µM, respectively (Hong et al., 2022). To determine a sublethal concentration, *E. coli* cells were treated with various concentrations of LNT113, and bacterial turbidity was measured for 60 min (Fig. 1A). LNT113 at 0.16 and 0.32 µM resulted in moderate turbidity reduction without inducing immediate bacteriolysis, indicating that these concentrations fall within the subinhibitory range. In contrast, LNT113 at 0.64 µM and higher concentrations caused a rapid reduction in turbidity upon treatment, suggesting bactericidal activity. Bacterial viability showed approximately 1.6-log decrease after 15 min LNT113 treatments at 0.64 µM (Fig. 1B). Based on these observations, *E. coli* cells were treated with 0.64 µM LNT113 for 15 min to represent sublethal LNT113 stress.

### Overview of transcriptional profiles of LNT113-treated *E. coli* cells

Transcriptomic analysis was performed to investigate the physiological responses of *E. coli* MG1655 following treatment with 0.64 µM LNT113 for 15 min. The experiment was designed with two comparative conditions: T1 (Tris buffer, 15 min vs. Tris buffer, 0 min) to assess the baseline effect of the buffer, and T2 (LNT113, 15 min vs. Tris buffer, 15 min) to evaluate the specific impact of LNT113. A total of 552 DEGs were identified after LNT113 treatment under the T2 condition, based on a threshold of log_2_ fold change ≥ 1 or ≤ –1. Among these, the expressions of 339 genes were upregulated (log_2_ fold change ≥ 1), and those of 213 genes were downregulated (log_2_ fold change ≤ –1). These DEGs were categorized into 34 and 24 functional groups according to their predicted functions based on KEGG and COG analyses, respectively (Fig. S3). Endolysins act externally to degrade the bacterial peptidoglycan layer (Fischetti, 2005), leading to cell wall damage that may trigger structural repair responses and metabolic reprogramming in bacterial cells (Peng et al., 2025). Accordingly, 222 genes associated with cell damage repair and metabolic adaptation to stress were prioritized from the total set of 552 DEGs for further analysis. To refine the list of candidate DEGs, 54 downstream genes within operons were excluded to avoid redundancy, as their expression patterns were likely to mirror those of upstream genes. Consequently, 168 representative DEGs were retained for further analysis. The expression patterns of the selected 168 DEGs were compared based on RNA-seq data and visualized using heatmaps (Fig. 2). These genes were classified into 11 representative functional categories, comprising six categories from KEGG and five from COG. Among these, 147 DEGs were shared between the two databases, whereas 11 were unique to KEGG and 10 were unique to COG. The expressions of genes associated with amino acid metabolism (K1) and lipid metabolism (K2) showed 84% upregulation and 16% downregulation. Genes related to carbohydrate metabolism (K3) exhibited 54% upregulation and 46% downregulation in their expressions, whereas those involved in membrane transport (K4) showed 73% upregulation and 27% downregulation in their expressions. Signal transduction-related genes (K5) displayed 62.5% upregulation and 37.5% downregulation in their expressions, whereas genes encoding protein families associated with signaling and cellular processes (K6) showed 49% upregulation and 51% downregulation in their expressions. The expressions of genes involved in cell wall/membrane/envelope biogenesis (C1) exhibited 43% upregulation and 57% downregulation. The expression of *lepB*, associated with intracellular trafficking, secretion, and vesicular transport (C2), was downregulated. In contrast, the expressions of genes associated with cell motility (C3) and glycan biosynthesis and metabolism (C5) showed 100% upregulation. The expressions of genes related to cellular community (C4) exhibited 25% upregulation and 75% downregulation (Fig. 2).

### Exploring the genetic determinants associated with bacterial tolerance to LNT113

To validate the transcriptomic results, the mRNA expression levels of the selected 168 genes were analyzed using RT-qPCR (Fig. S4) and the expression patterns were visually compared with RNA-seq data using heatmaps (Fig. S5). Most genes showed comparable expression patterns between two experimental approaches under T2 conditions. In order to cancel out the bacterial response to the buffer only, transcription levels were compared between Tris buffer (15 min) and LNT113 (15 min) conditions and the genes with significant difference in their transcription between two conditions were determined (*p* < 0.05). In the comparison with the Tris-buffered condition, the expressions of eight genes were significantly upregulated and those of five were significantly downregulated in response to LNT113 treatment (Fig. 3A; yellow-highlighted genes in Figs. S4 and S5). The genes with upregulated expressions—*aspC* (aspartate aminotransferase), *aroC* (chorismate synthase), *fabB* (β-ketoacyl-[acyl carrier protein] synthase I), *glnA* (glutamine synthetase), *prpB* (2-methylisocitrate lyase), *nanC* (N-acetylneuraminate outer membrane channel), *phoE* (outer membrane porin PhoE), and *kdpB* (K^+^-transporting P-type ATPase subunit KdpB)—and those with downregulated expressions—*cusC* (copper/silver export system outer membrane channel), *ddpX* (D-alanyl-D-alanine dipeptidase), *fdnI* (formate dehydrogenase N subunit gamma), *bssS* (regulator of biofilm formation), and *araF* (arabinose ABC transporter periplasmic binding protein)—were selected for further analysis.

The 13 genes that showed significant expression changes upon LNT113 exposure may be associated with bacterial tolerance to endolysin stress. To assess their potential contribution to bacterial adaptation to endolysin treatment, each of the 13 genes was individually deleted, and the susceptibility of each mutant strain to LNT113 was analyzed. When a candidate gene was part of an operon, the entire operon was deleted because genes within the same operon are frequently co-transcribed and co-regulated and function cooperatively within a common pathway or cellular process (Bratlie et al., 2010). The deleted operons in the 13 mutants are listed in Table 1. In the absence of LNT113, most mutants exhibited growth patterns comparable to that of the wild-type. strain, except for Δ*aspC*, which showed a growth defect (data not shown). To evaluate the impact of specific candidate gene deletions on bacterial susceptibility to LNT113, an OD_600_ reduction assay was conducted using the 13 mutants and the *E. coli* wild-type strain (Fig. 3B and 3C). This analysis revealed distinct susceptibility patterns among the gene deletion mutants in both logarithmic and stationary growth phases. Δ*fabB* and Δ*prmB*–*yfcL* exhibited markedly greater OD_600_ reductions in both growth phases, indicating increased vulnerability to LNT113. Except for these two mutants, most mutants displayed OD_600_ changes comparable to those of the wild type. These results suggest that the *prmB*–*yfcL* operon and *fabB* play protective roles during endolysin-induced stress.

### Identification of genes critical for bacterial tolerance to LNT113

Given their susceptibility phenotypes and biological relevance, the *fabB* and the *prmB*–*yfcL* operon were further examined to determine whether their deletion compromises membrane permeability under endolysin-induced stress. NPN, which fluoresces upon insertion into disrupted membranes, was used as a probe to assess membrane permeability following LNT113 treatment and to compare responses among deletion mutants. Compared with the wild-type *E. coli* MG1655 strain, the Δ*fabB* and Δ(*prmB*–*yfcL*) mutants exhibited increased NPN uptake following treatment with 0.64 µM LNT113, indicating compromised membrane integrity (Fig. 4A and 4B). These two mutants demonstrated comparable NPN uptake levels to those of the wild-type strain in the absence of LNT113 (Fig. S6A), suggesting that *fabB* and *prmB*–*yfcL* operon contribute to maintaining membrane integrity under endolysin-induced stress. The Δ(*prmB*–*yfcL*) strain lacks six genes: *prmB*, *aroC*, *mepA*, *yfcA*, *epmC*, and *yfcL*. To determine which gene is essential for bacterial tolerance to LNT113, individual deletion mutants were constructed for each gene in the operon, and their susceptibility to LNT113 was examined. The NPN uptake assay revealed that deletion of *aroC* and *mepA* resulted in increased membrane permeability compared with that of the wild-type strain (Fig. 4C and 4D), whereas the other single-gene deletions showed minimal effects (Fig. S6B). These findings indicate that *fabB*, *aroC*, and *mepA* are critical determinants for maintaining bacterial membrane integrity under LNT113-induced endolysin stress.

To determine whether the increased membrane permeability observed in the Δ*fabB*, Δ*aroC*, and Δ*mepA* mutants resulted from the loss of each gene itself or from potential polar effects, complementation assays were performed by introducing plasmids carrying the respective genes into the deletion mutants. The *fabB*, *aroC*, and *mepA* genes were cloned into the low-copy plasmid pWSK129 under their native promoters, and the resulting constructs—pWSK129::*fabB*, pWSK129::*aroC*, and pWSK129::*mepA*—were subsequently introduced into the corresponding deletion mutants. Membrane permeability of the complemented strains was then evaluated using NPN uptake assays under the same conditions (Fig. 5). Expression of *fabB* and *mepA* from pWSK129::*fabB* and pWSK129::*mepA*, respectively, restored membrane integrity in the Δ*fabB* and Δ*mepA* mutants to wild-type levels, demonstrating successful functional complementation. In contrast, complementation of Δ*aroC* with pWSK129::*aroC* resulted in partial restoration of membrane integrity, suggesting that the envelope stress associated with *aroC* deletion may arise from broader metabolic disturbances rather than from direct structural defects.

## Discussion

The development of recombinant endolysins as alternative antimicrobial agents represents a promising strategy to combat antibiotic-resistant pathogens and reduce dependence on conventional antibiotic therapy (Tesema, 2025). Previous studies have demonstrated that the engineered endolysin LNT113, generated by fusing cecropin A to the parental endolysin, exhibits marked bactericidal efficacy against Gram-negative bacteria despite the presence of the outer membrane (Hong et al., 2022). LNT113 displays enhanced outer membrane permeabilization, increased antimicrobial activity, and synergistic effects in combination with conventional antibiotics (Cho et al., 2024; Hong et al., 2022). Building upon these findings, the present study focused on bacterial transcriptional responses induced by sublethal exposure to LNT113 to better understand its impact on cellular physiology beyond direct bacteriolytic activity. The schematic analysis flow is drawn in Fig. S7. Comparative transcriptomic analysis was conducted under T1 (Tris buffer, 15 min vs. Tris buffer, 0 min) and T2 (LNT113, 15 min vs. Tris buffer, 15 min) conditions. A total of 552 DEGs were selected from T2 condition and then narrowed down to 168 DEGs, prioritizing those associated with cell damage repair and metabolic adaptation to stress. The set of 168 DEGs were subjected to RT-qPCR under Tris buffer (15 min) and LNT113 (15 min) conditions and thirteen DEGs with different transcription levels between two conditions (*p* < 0.05) were finally chosen as candidate resistance determinants against endolysin LNT113. Bacterial mutant strains lacking candidate DEGs were constructed and their phenotypic characteristics were investigated to determine the genes critical for bacterial tolerance to LNT113.

Previous studies have demonstrated that subinhibitory exposure to endolysins can induce significant transcriptional reprogramming. Subinhibitory doses of CHAPSH3b were reported to decrease biofilm formation in *Staphylococcus aureus* by downregulating the expressions of autolysin-associated genes (Fernandez et al., 2017). Similarly, another study showed that sublethal treatment with a recombinant endolysin stimulated an adherent-invasive *E. coli* strain to upregulate the expressions of genes involved in flagellar biosynthesis (Hwang et al., 2022). Our transcriptomic analysis identified 552 DEGs following sublethal LNT113 exposure, with a predominance of genes with upregulated expressions, suggesting extensive transcriptional reprogramming in response to endolysin-induced stress. Together, these findings support the notion that endolysin exposure at sublethal concentrations can reprogram bacterial gene expression and alter physiological behavior. Considering that endolysins compromise cell envelope integrity via peptidoglycan degradation, genes associated with envelope repair and metabolic adaptation may be required for bacterial adaptation to sublethal endolysin stress. Therefore, 168 DEGs relevant to envelope biogenesis and repair were prioritized to identify resistance determinants against endolysins. Among these, deletion of *fabB* and *prmB*–*yfcL* attenuated bacterial growth under LNT113 treatment, indicating that these genes are important for resistance to sublethal endolysin stress. The NPN uptake assay revealed that *E. coli* strains lacking *fabB* and *prmB*–*yfcL* exhibited increased membrane permeability.

The *fabB* gene encodes β-ketoacyl-acyl carrier protein synthase I (KAS I), which is essential for the biosynthesis of unsaturated fatty acids and plays a critical role in maintaining membrane lipid composition and stability (Feng and Cronan, 2009). It is speculative that deletion of *fabB* likely reduces unsaturated fatty acid biosynthesis, thereby altering membrane fluidity and perturbing lipid homeostasis. Changes in membrane lipid composition have been shown to influence cellular physiology (Budin et al., 2018). Altered membrane rigidity may consequently affect outer membrane organization and permeability, potentially facilitating LNT113 access to the periplasmic peptidoglycan layer. The increased NPN uptake and turbidity reduction observed in the Δ*fabB* mutant highlight the importance of *fabB* in maintaining envelope stability. The upregulation of its expression under endolysin stress may represent a protective cellular response aimed at preserving membrane fluidity and limiting LNT113 access to the peptidoglycan layer.

The *prmB*–*yfcL* operon consists of six genes with diverse roles in translation regulation, amino acid metabolism, and cell envelope maintenance. *prmB* encodes a ribosomal protein L3 methyltransferase that contributes to ribosome function and translation efficiency (Colson et al., 1979); *aroC* encodes chorismate synthase, a key enzyme in the shikimate pathway responsible for producing chorismate, a central precursor of aromatic amino acids and related metabolites (White et al., 1988); *mepA* encodes a murein endopeptidase involved in peptidoglycan remodeling (Marcyjaniak et al., 2004); and *epmC* encodes a protein involved in elongation factor P modification, which is essential for efficient translation elongation (Peil et al., 2012). Additionally, *yfcA* is predicted to encode a periplasmic binding protein, whereas *yfcL* is annotated as a putative transporter or stress-related membrane protein. When each gene within the *prmB*–*yfcL* operon was deleted to differentiate the roles of individual genes, the Δ*aroC* and Δ*mepA* mutants showed increased membrane permeability and reduced survival under LNT113 treatment, highlighting their presumptive roles in maintaining cellular integrity under stress. The upregulation of *aroC* expression under endolysin-induced stress suggests enhanced activity of the shikimate pathway, potentially contributing to metabolic adaptation and redirection of biosynthetic flux. Aromatic amino acids produced via the shikimate pathway, such as phenylalanine, tyrosine, and tryptophan, are essential for protein synthesis and cellular stress responses (Li et al., 2022). Because metabolic pathway reorganization plays a central role in stress adaptation in *E. coli* (Du et al., 2019), increased aromatic amino acid biosynthesis may provisionally support adaptive metabolic remodeling under endolysin-induced stress. Given its role in aromatic amino acid biosynthesis, *aroC* may indirectly contribute to envelope stability by modulating metabolic responses during stress adaptation. In parallel, increased *mepA* expression under endolysin-induced stress is suggestive of activation of peptidoglycan remodeling pathways. As a murein endopeptidase involved in peptidoglycan turnover (Keck et al., 1990), *mepA* may contribute to maintaining envelope integrity and mitigating cell wall damage during LNT113 exposure.

Although the effects of cecropin A–mediated outer membrane permeabilization cannot be entirely separated from the intrinsic activity of LNT113, the present study provides comprehensive transcriptomic and phenotypic insights into bacterial responses under sublethal endolysin treatment. Understanding the transcriptional and phenotypic responses induced by LNT113 may facilitate the development of combination therapies and guide dose optimization strategies to minimize adaptive stress responses, thereby enhancing endolysin efficacy against MDR Gram-negative pathogens.

## Figures and Tables

**Fig. 1.**
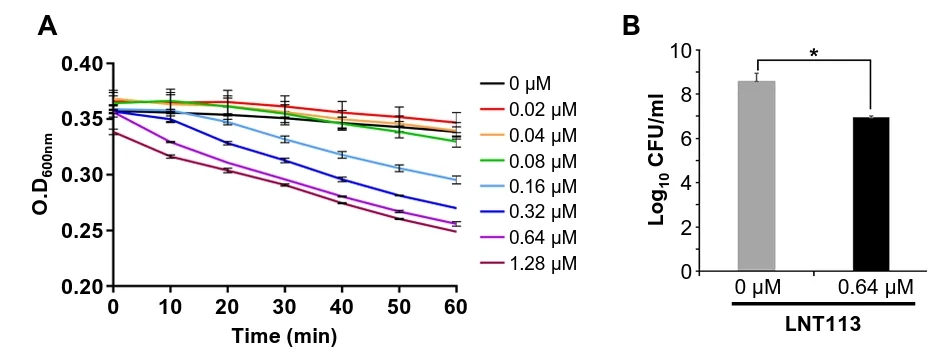
Antibacterial activity of engineered endolysin LNT113. (A) *Escherichia coli* MG1655 (4 × 10^9^ cells) was treated with different concentrations of LNT113 ranging from 0 to 1.28 μM. Bacterial turbidity was measured for 60 min and plotted over time. (B) *E. coli* MG1655 cells were treated with 0.64 μM LNT113 for 15 min and the numbers of live cells were counted. Significant difference was denoted with an asterisk (*p* < 0.05). Experiments were performed in triplicate. Data are expressed as the Mean ± standard deviation.

**Fig. 2.**
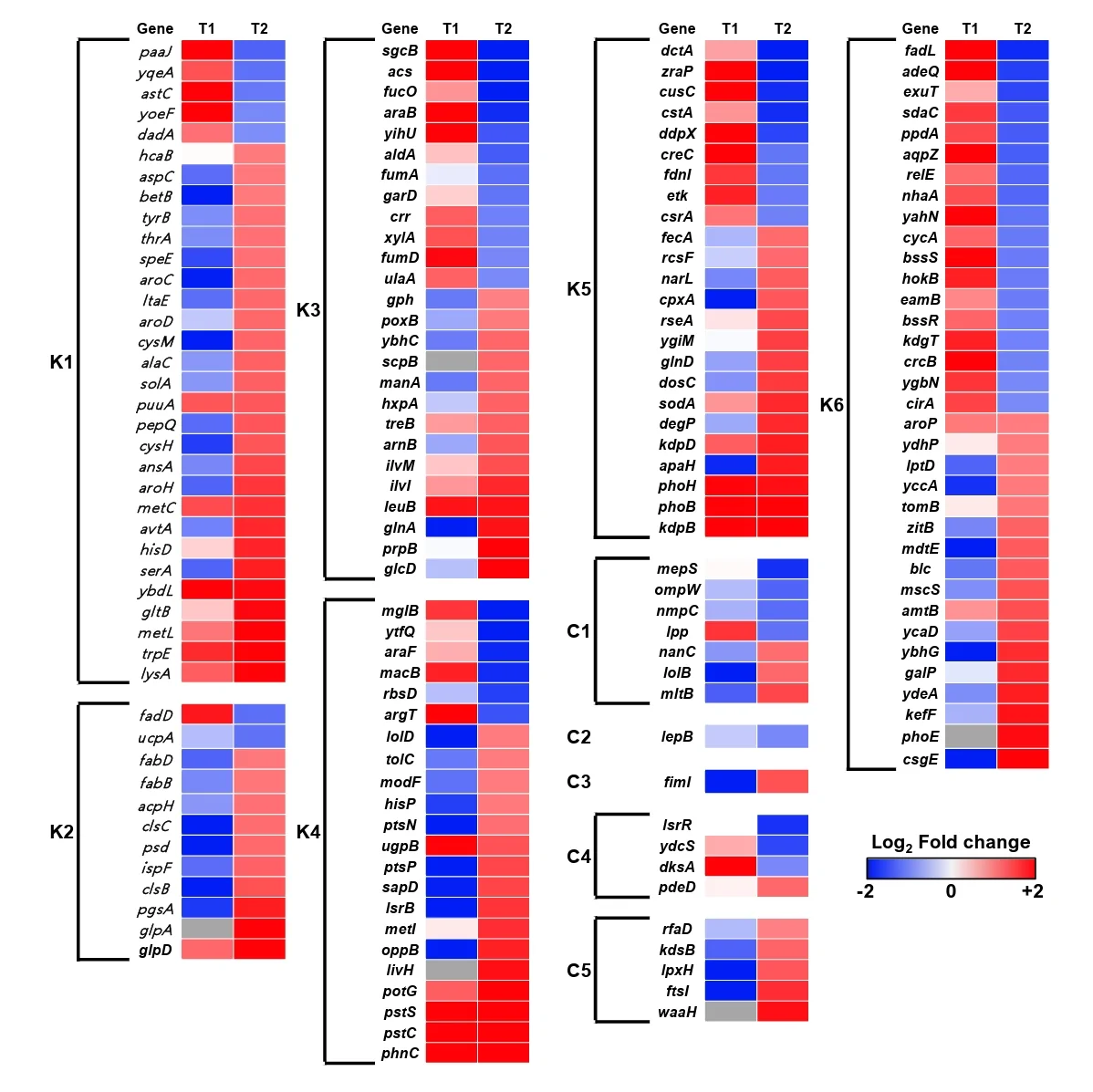
Comparative transcriptome analysis of *Escherichia coli* following LNT113 treatment. Genes were categorized according to the Kyoto Encyclopedia of Genes and Genomes (KEGG) and Clusters of Orthologous Groups (COG) functional classifications. Genes identified in both KEGG and COG were annotated according to KEGG. KEGG categories: K1, amino acid metabolism; K2, lipid metabolism; K3, carbohydrate metabolism; K4, membrane transport; K5, signal transduction; K6, protein families—signaling and cellular processes. COG categories: C1, cell wall/membrane/envelope biogenesis; C2, intracellular trafficking, secretion, and vesicular transport; C3, cell motility; C4, cellular community; C5, glycan biosynthesis and metabolism. Experimental conditions: T1, Tris buffer, 15 min vs. Tris buffer, 0 min; T2, LNT113, 15 min vs. Tris buffer, 15 min. Log2 fold change values were calculated in T1 and T2 conditions using transcripts per million (TPM) ratios and displayed in heatmaps. Gray indicates “not determined" due to the lack of TPM values.

**Fig. 3.**
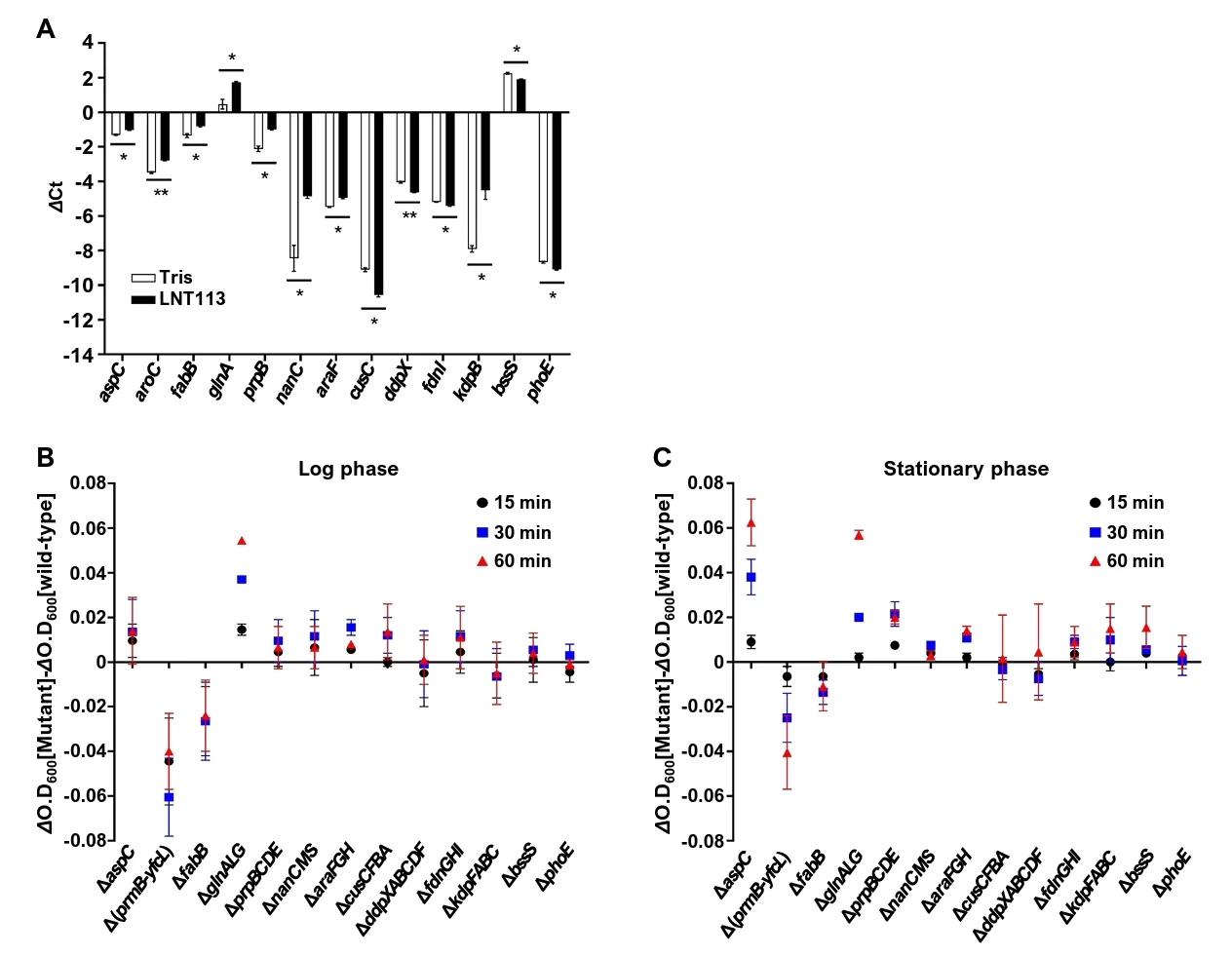
Bacterial susceptibility to LNT113 in the absence of 13 candidate genes. (A) Quantitative reverse transcription polymerase chain reaction (RT-qPCR) analysis of candidate genes potentially associated with endolysin resistance. The expression levels of each gene were normalized to *rpoD* under Tris buffer (15 min) and LNT113 (0.64 µM, 15 min) conditions, and ΔCt values were presented as the Mean ± standard error of the mean (SEM). RT-qPCR was conducted at least three times, using separately harvested RNAs, and asterisks indicate statistically significant differences between two conditions (*, *p* < 0.05; **, *p* < 0.01). (B, C) OD_600_ reduction assays comparing the susceptibility of deletion mutants with the wild-type strain in the logarithmic growth phase (B) and stationary growth phase (C). Bacterial cells were treated with 0.64 μM LNT113, and turbidity was measured at 15, 30, and 60 min. Differences in bacterial turbidity between each mutant and the wild-type strain are presented as vertically stacked values. Experiments were performed in triplicate and the result is displayed by the Mean ± SEM.

**Fig. 4.**
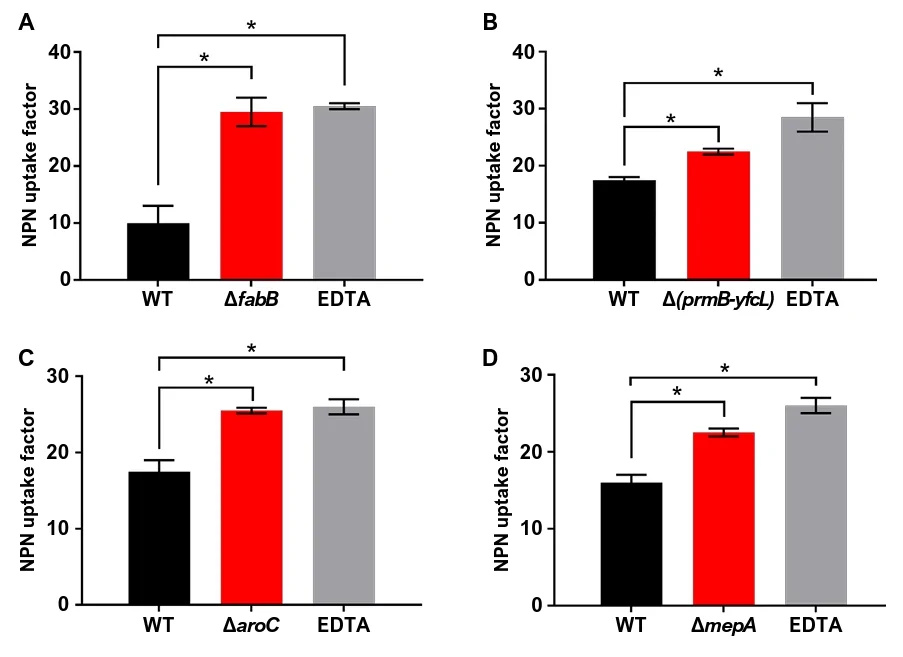
Comparison of NPN uptake in bacteria lacking candidate genes. Bacterial membrane permeability was assessed in Δ*fabB* (A), Δ*prmB*–*yfcL* (B), Δ*aroC* (C), and Δ*mepA* (D). Bacterial cells were treated with 0.64 μM LNT113 for 5 min. Negative control: wild-type *Escherichia coli* MG1655; positive control: 1 mM EDTA-treated *E. coli* MG1655 cells. Data represent the Mean ± standard error of the mean from three biological replicates. Statistically significant differences (*p* < 0.05) are denoted by asterisks.

**Fig. 5.**
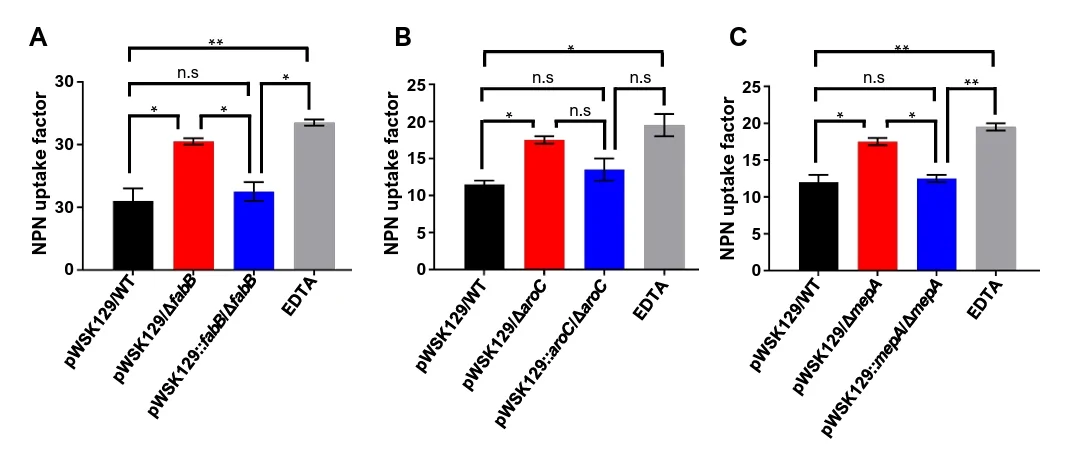
Comparison of NPN uptake in gene-complemented mutants and wild-type *Escherichia coli*. Bacterial membrane permeability of Δ*fabB*, Δ*aroC*, and Δ*mepA* strains complemented with pWSK129::*fabB* (A), pWSK129::*aroC* (B), and pWSK129::*mepA* (C), respectively, was compared with that of wild-type *E. coli* MG1655 carrying the empty vector pWSK129. Bacterial cells were treated with 0.64 μM LNT113, and the NPN uptake assay was performed after 5 min. EDTA-treated mutant cells were used as controls. Data represent the Mean ± standard error of the mean from three biological replicates. An asterisk indicates a statistically significant difference: *p < 0.05, **p < 0.01.

**Table 1.** Thirteen mutant strains lacking potential tolerance-associated genes

Group	Gene^*^	Function	Mutant
Amino acid metabolism	*aspC*	Aspartate aminotransferase	Δ*aspC*
	*prmB*	50S ribosomal subunit protein L3 N(5)-glutamine methyltransferase	Δ(*prmB*–*yfcL*)
	*aroC*	Chorismate synthase	
	*mepA*	Peptidoglycan DD-endopeptidase/peptidoglycan LD-endopeptidase	
	*epmC*	EF-P Lys34 hydroxylase	
	*yfcA*	Conserved inner membrane protein YfcA	
	*yfcL*	Protein YfcL	
Lipid metabolism	*fabB*	β-Ketoacyl-[acyl carrier protein] synthase I	Δ*fabB*
Carbohydrate metabolism	*glnA*	Glutamine synthetase	Δ*glnALG*
	*glnL*	Sensory histidine kinase	
	*glnG*	DNA-binding transcriptional dual regulator	
	*prpB*	2-Methylisocitrate lyase	Δ*prpBCDE*
	*prpC*	2-Methylcitrate synthase	
	*prpD*	2-Methylcitrate dehydratase	
	*prpE*	Propionyl-CoA synthetase	
Membrane transport	*araF*	Arabinose ABC transporter periplasmic binding protein	Δ*araFGH*
	*araG*	Arabinose ABC transporter ATP-binding subunit	
	*araH*	Arabinose ABC transporter membrane subunit	
Signal transduction	*cusC*	Copper/silver export system outer membrane channel	*ΔcusCFBA*
	*cusF*	Copper/silver export system periplasmic binding protein	
	*cusB*	Copper/silver export system membrane fusion protein	
	*cusA*	Copper/silver export system RND permease	
	*ddpX*	D-Alanyl-D-alanine dipeptidase	Δ*ddpXABCDF*
	*ddpA*	Putative D,D-dipeptide ABC transporter periplasmic binding protein	
	*ddpB*	Putative D,D-dipeptide ABC transporter membrane subunit	
	*ddpC*	Putative D,D-dipeptide ABC transporter membrane subunit	
	*ddpD*	Putative D,D-dipeptide ABC transporter ATP-binding subunit	
	*ddpF*	Putative D,D-dipeptide ABC transporter ATP-binding subunit	
	*fdnG*	Formate dehydrogenase N subunit alpha	Δ*fdnGHI*
	*fdnH*	Formate dehydrogenase N subunit beta	
	*fdnI*	Formate dehydrogenase N subunit gamma	
	*kdpF*	K^+^-transporting P-type ATPase subunit	Δ*kdpFABC*
	*kdpA*	K^+^-transporting P-type ATPase subunit	
	*kdpB*	K^+^-transporting P-type ATPase subunit	
	*kdpC*	K^+^-transporting P-type ATPase subunit	
Protein families: signaling and cellular processes	*bssS*	Regulator of biofilm formation	Δ*bssS*
	*phoE*	Outer membrane porin PhoE	Δ*phoE*
Cell wall/membrane/envelope biogenesis	*nanC*	N-Acetylneuraminate outer membrane channel	Δ*nanCMS*
	*nanM*	N-Acetylneuraminate mutarotase	
	*nanS*	N-Acetyl-9-O-acetylneuraminate esterase	

* Thirteen candidate genes with significant expression changes following LNT113 treatment are highlighted in red.

## Supplementary Information

Table S1. Bacterial strains used in this study

Table S2. Primers used in strain construction

Table S3. Primers used in plasmid construction

Table S4. Primer used in RT-qPCR

Fig. S1. Purification of endolysin LNT113. Purified LNT113 was loaded onto a 10% SDS–PAGE gel. Lane 1, whole-cell lysate; lane 2, pellet fraction after bacterial lysis; lane 3, purified protein; lane M, molecular weight marker.

Fig. S2. Principal component analysis (PCA) of RNA sequencing results. PCA showed tight clustering of biological replicates, depending on the RNA preparation conditions. Samples from the Tris buffer 15 min and LNT113 15 min were clearly separated from those of the Tris buffer 0 min control, indicating high reproducibility and treatment-dependent transcriptional differences.

Fig. S3. Functional categorization of genes with altered mRNA levels following LNT113 treatment. Differentially expressed genes (DEGs) were grouped based on (A) Kyoto Encyclopedia of Genes and Genomes (KEGG) classification and (B) Clusters of Orthologous Groups (COG) analysis. Red and blue bars represent the percentages of genes with upregulated and downregulated expressions, respectively, in each functional category.

Fig. S4. Quantification of mRNA levels of selected differentially expressed genes (DEGs) using quantitative reverse transcription polymerase chain reaction (RT-qPCR). Relative mRNA levels were calculated under Tris buffer (15 min) and LNT113 (0.64 μM, 15 min) conditions. Ct values for each gene were normalized to *rpoD*, and relative expression levels are presented as ΔCt values in T (Tris buffer 15 min) and L (LNT113 15 min) columns. Yellow-highlighted genes exhibited statistically significant differences between two conditions (*p* < 0.05).

Fig. S5. Comparison of expression patterns between RNA-seq and RT-qPCR. Heat maps illustrate the expression profiles of 168 differentially expressed genes (DEGs) determined by RNA-seq (left column of each pair) and RT-qPCR (right column of each pair). Genes are organized according to their KEGG and COG functional categories, with each row corresponding to a single gene. Expression changes induced by LNT113 treatment are represented by color intensity, where red denotes upregulated genes and blue denotes downregulated genes. RT-qPCR expression levels are reported as ΔΔCt values, calculated by subtracting the ΔCt of the control group (Tris buffer, 15 min) from that of the LNT113-treated group (15 min), with ΔCt defined as Ct_[*rpoD*]_ – Ct_[target gene]_ (n = 2 per group). RNA-seq results are shown as fold changes between the LNT113-treated (15 min) and control (Tris buffer, 15 min) samples. Genes highlighted in yellow exhibited statistically significant differences in RT-qPCR analysis (*p* < 0.05) and were subsequently selected for functional validation.

Fig. S6. Comparison of NPN uptake in bacteria lacking candidate genes. (A) Membrane permeability was assessed by the NPN uptake assay in *Escherichia coli* MG1655 wild-type, Δ*fabB*, Δ(*prmB*–*yfcL*), Δ*aroC*, Δ*mepA*, Δ*prmB*, Δ*yfcA*, Δ*epmC*, and Δ*yfcL* strains in the absence of LNT113 treatment. (B) Bacterial membrane permeability was assessed in Δ*prmB*, Δ*yfcA*, Δ*epmC*, and Δ*yfcL*. Bacterial cells were treated with 0.64 μM LNT113 for 5 min. Negative control: wild-type *Escherichia coli* MG1655; positive control: 1 mM EDTA-treated *E. coli* MG1655 cells. Data represent the Mean ± standard error of the mean from three biological replicates. Statistically significant differences (*p* < 0.05) are denoted by asterisks.

Fig. S7. Schematic analytical flow. ① Comparative transcriptomic analysis was conducted under T1 (Tris buffer, 15 min vs. Tris buffer, 0 min) and T2 (LNT113, 15 min vs. Tris buffer, 15 min) conditions. ② A total of 552 DEGs were selected from T2 condition and ③ then narrowed down to 168 DEGs, prioritizing those associated with cell damage repair and metabolic adaptation to stress. ④ The set of 168 DEGs were subjected to RT-qPCR under Tris buffer (15 min) and LNT113 (15 min) conditions and thirteen DEGs with different transcription levels between two conditions (*p* < 0.05) were finally chosen as candidate resistance determinants against endolysin LNT113. ⑤ Bacterial mutant strains lacking candidate DEGs were constructed and their phenotypic characteristics were investigated to determine the genes critical for bacterial tolerance to LNT113.
