# Supplementary material for: Transcriptomic insights into the effects of sublethal exposure to endolysin LNT113 on Escherichia coli
Source: J Microbiol. 2026 Jul 31;64(7):e2605009. doi: 10.71150/jm.2605009 (PMC13714653; doi:10.71150/jm.2605009)
Supplement: Supplementary file 1 [file jm-2605009-Supplementary-Fig-S1.pdf]

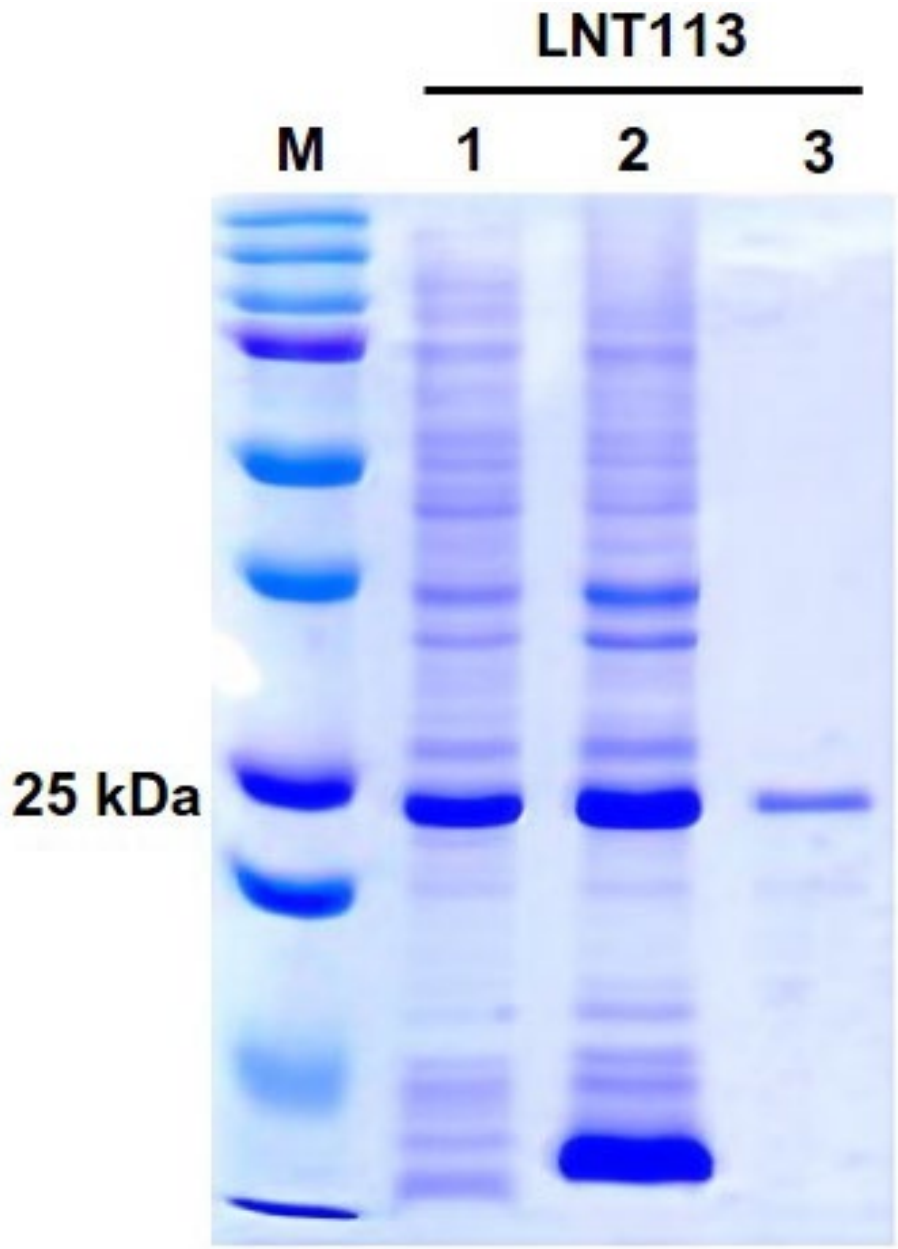

Fig. S1. Purification of endolysin LNT113. Purified LNT113 was loaded onto a 10% SDS–PAGE gel. Lane 1, whole-cell lysate; lane 2, pellet fraction after bacterial lysis; lane 3, purified protein; lane M, molecular weight marker.

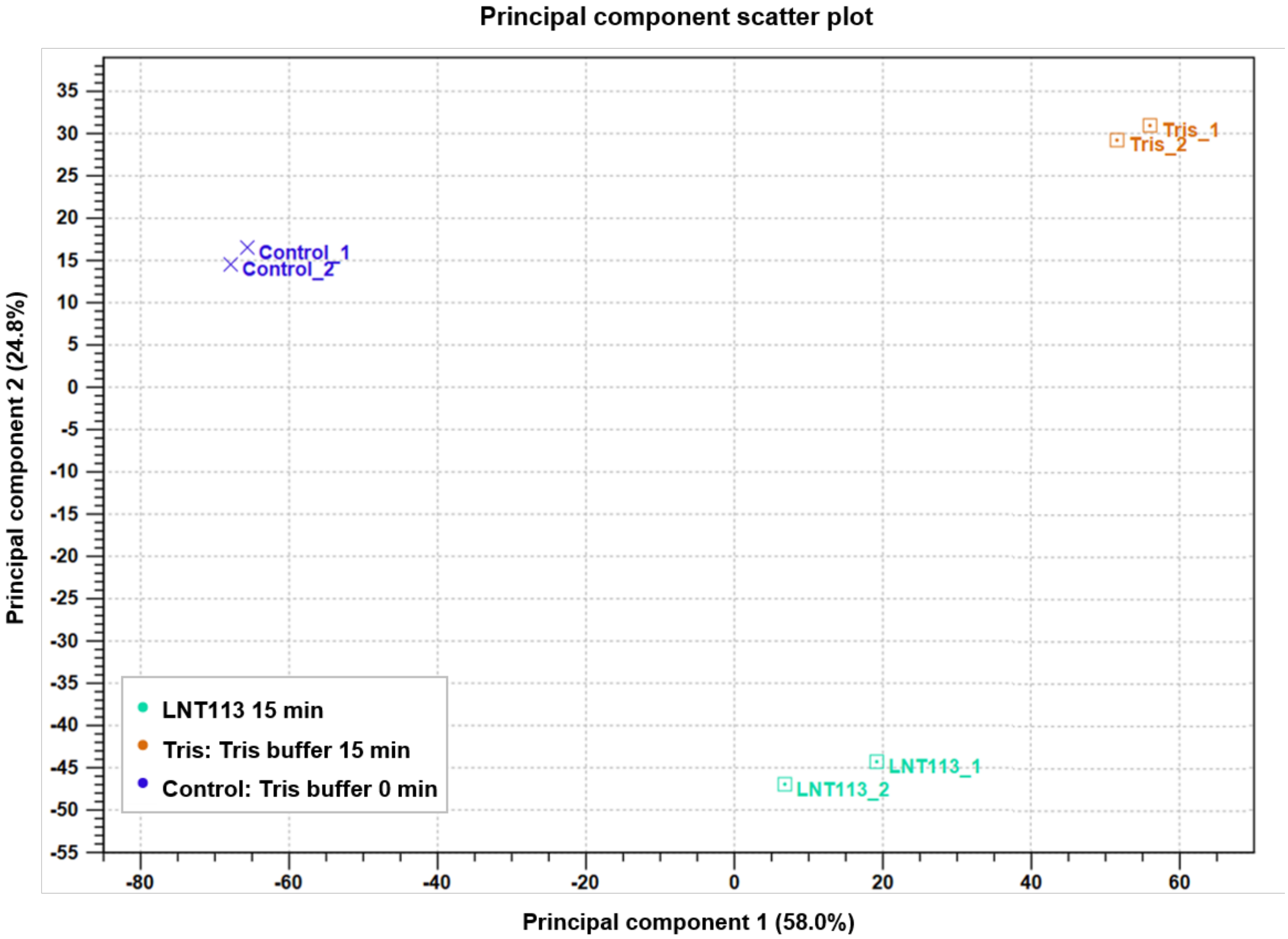

Fig. S2. Principal component analysis (PCA) of RNA sequencing results. PCA showed tight clustering of biological replicates, depending on the RNA preparation conditions. Samples from the Tris buffer 15 min and LNT113 15 min were clearly separated from those of the Tris buffer 0 min control, indicating high reproducibility and treatment-dependent transcriptional differences.

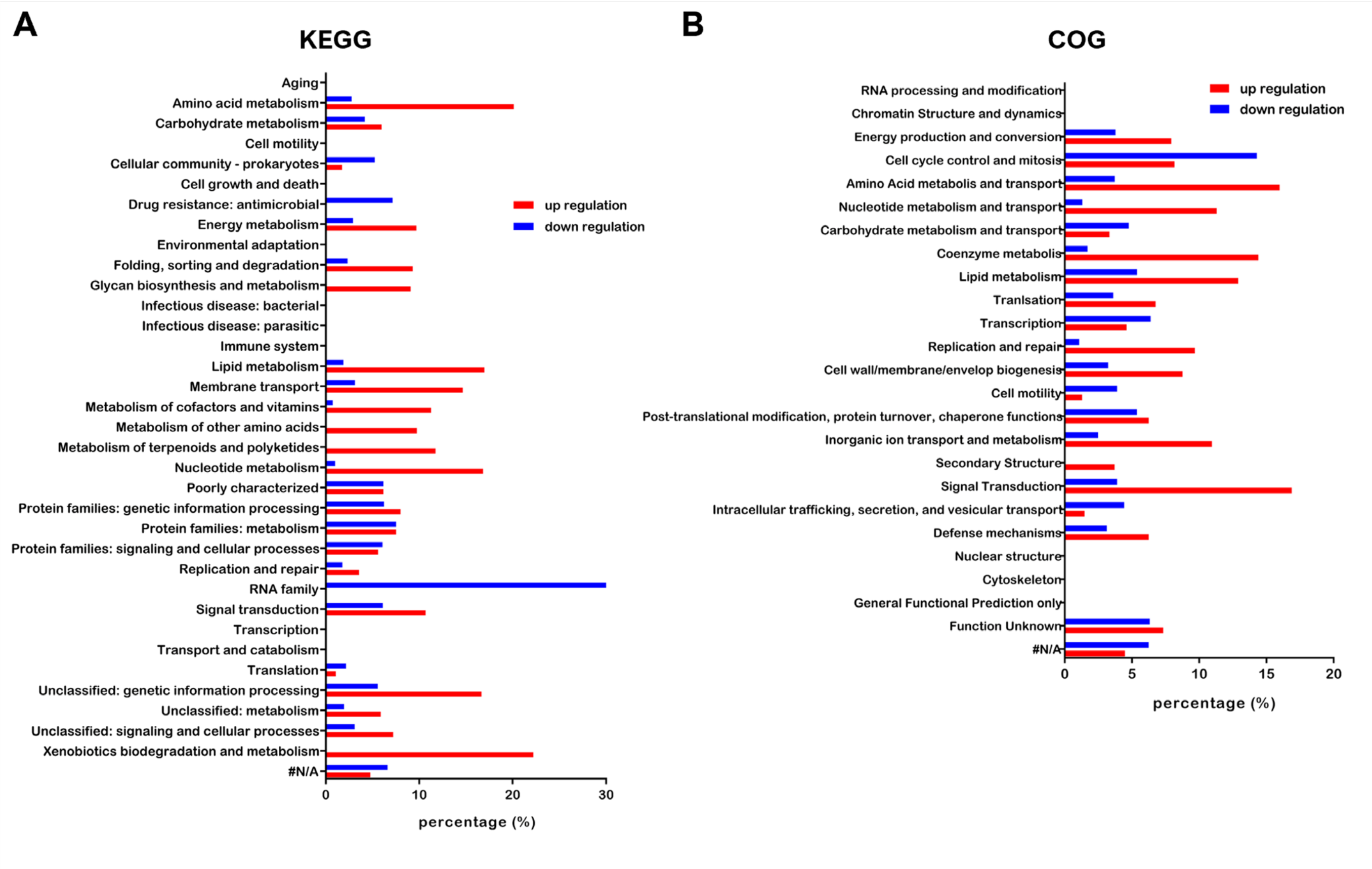

Fig. S3. Functional categorization of genes with altered mRNA levels following LNT113 treatment. Differentially expressed genes (DEGs) were grouped based on (A) Kyoto Encyclopedia of Genes and Genomes (KEGG) classification and (B) Clusters of Orthologous Groups (COG) analysis. Red and blue bars represent the percentages of genes with upregulated and downregulated expressions, respectively, in each functional category.

Cluster	Gene	T	L	p-value
	paaJ	-6.0	-5.6	0.32
	paaK	-3.8	-3.1	0.35
	yqeA	-5.4	-5.1	0.37
	astC	-1.0	-0.8	0.62
	yoeF	-5.8	-5.5	0.42
	dadA	0.5	0.2	0.59
	hcaB	-4.9	-5.2	0.13
	aspC	-1.3	-1.0	0.03
	betB	-3.7	-3.5	0.67
	tyrB	-2.0	-2.2	0.48
	thrA	-2.8	-2.3	0.21
	speE	-1.3	-1.2	0.81
	aroC	-3.5	-2.8	0.04
	ltaE	-1.2	-1.0	0.84
	aroD	-3.1	-3.1	0.89
	cysM	-2.1	-2.0	0.62
	alaC	-3.5	-3.6	0.43
K1	solA	-5.1	-4.8	0.21
	puuA	-4.5	-3.2	0.06
	pepQ	-2.1	-1.8	0.11
	cysH	-1.4	-1.1	0.15
	metA	-1.5	-0.9	0.34
	ansA	-3.4	-3.9	0.29
	aroH	-2.3	-2.3	0.98
	metC	-4.1	-3.8	0.66
	avtA	-2.7	-2.5	0.82
	hisD	-1.0	-0.5	0.27
	serA	-0.7	0.0	0.08
	ybdL	-2.4	-1.8	0.48
	gltB	-0.4	-0.2	0.64
	metL	-2.2	-1.9	0.28
	trpE	1.8	2.2	0.18
	hisB	-2.0	-2.0	0.84
	lysA	-7.8	-5.9	0.06
	trpB	0.3	0.2	0.89
	fadD	0.9	0.3	0.08
	ucpA	-1.9	-2.0	0.81
	fabD	-0.5	-0.5	0.93
	fabB	-1.4	-0.8	0.04
	acpH	-4.5	-4.4	0.29
K2	clsC	-3.5	-3.3	0.51
	psd	-3.3	-2.8	0.19
	ispF	-3.8	-3.6	0.59
	clsB	-4.7	-4.6	0.87
	pgsA	-4.2	-3.8	0.23
	glpA	-8.2	-6.6	0.36
	glpD	-3.8	-2.6	0.18

Cluster	Gene	T	L	p-value
	sgcB	-5.2	-5.1	0.66
	yqeF	-3.1	-2.8	0.26
	acs	-0.2	-0.2	0.95
	fucO	-4.1	-3.8	0.36
	araB	-8.4	-8.3	0.56
	yihU	-8.7	-8.7	0.99
	aldA	0.7	0.6	0.85
	fumA	-0.6	-0.6	0.94
	garD	-5.7	-5.7	0.88
	crr	1.5	1.8	0.35
	xylA	-6.1	-4.5	0.08
	fumD	-4.8	-4.3	0.20
	ulaA	-7.9	-7.5	0.30
K3	gph	-3.3	-3.1	0.24
	poxB	-1.5	-1.2	0.06
	ybhC	-5.1	-4.8	0.51
	scpB	-7.7	-7.4	0.66
	manA	-1.7	-1.7	0.89
	hxpA	-4.0	-3.7	0.39
	treB	-6.4	-6.2	0.50
	arnB	-5.1	-4.9	0.47
	ilvM	-4.8	-4.6	0.44
	ilvI	-5.5	-5.4	0.79
	treF	-2.0	-1.9	0.39
	leuB	-2.9	-2.8	0.77
	glnA	0.5	1.7	0.05
	prpB	-2.1	-1.0	0.02
	glcD	-1.4	-1.2	0.41
	mglB	-2.8	-3.4	0.17
	ytfQ	-2.6	-2.6	0.84
	araF	-5.5	-4.9	0.01
	macB	-5.1	-4.9	0.25
	rbsD	0.5	0.1	0.16
	argT	-1.2	-1.3	0.83
	lolD	-3.5	-3.2	0.32
	tolC	-1.7	-1.2	0.13
	modF	-5.9	-5.6	0.30
	hisP	-6.1	-5.9	0.84
	potB	-4.3	-4.0	0.26
	ptsN	-1.7	-1.4	0.47
K4	ugpB	-3.8	-3.8	0.97
	ptsP	-3.0	-2.8	0.31
	sapD	-2.9	-2.8	0.36
	lsrB	-2.9	-2.3	0.24
	metI	-3.3	-3.4	0.94
	oppB	-0.3	0.5	0.31
	livH	-7.2	-6.8	0.38
	livJ	-4.9	-4.8	0.30
	potG	-5.3	-5.3	0.95
	pstS	-1.9	-1.7	0.76
	pstC	-3.9	-3.8	0.93
	pstB	-3.3	-3.5	0.85
	phnC	-9.6	-9.1	0.36

Cluster	Gene	T	L	p-value
	dctA	-0.2	-0.1	0.78
	zraP	-6.8	-7.2	0.27
	cusC	-9.1	-10.6	0.01
	cstA	-1.7	-1.6	0.87
	ddpX	-4.0	-4.6	0.01
	creC	-5.5	-6.1	0.39
	fdnI	-5.2	-5.4	0.01
	etk	-5.9	-6.6	0.16
	csrA	2.1	2.1	0.95
	glnL	-4.3	-4.2	0.82
	fecA	-2.9	-3.0	0.85
	rcsF	-5.5	-5.3	0.50
K5	narL	-3.1	-3.2	0.81
	cpxA	-2.0	-1.8	0.24
	rseA	0.7	0.6	0.84
	ygiM	-2.9	-3.3	0.31
	glnD	-2.0	-2.0	0.68
	dosC	-2.0	-2.0	0.31
	sodA	-3.3	-2.9	0.54
	degP	-4.1	-3.7	0.28
	kdpD	-5.3	-5.4	0.75
	apaH	-4.4	-4.2	0.66
	phoH	-1.7	-1.0	0.26
	phoB	-3.5	-3.8	0.74
	kdpB	-7.9	-4.5	0.03

Cluster	Gene	T1	T2	p-value
	mepS	-8.7	-8.6	0.85
	ompW	0.2	0.2	0.73
	nmpC	-5.6	-5.5	0.95
C1	lpp	1.4	1.8	0.21
	nanC	-8.4	-4.9	0.04
	lolB	-4.4	-4.2	0.60
	mltB	-5.0	-4.8	0.54
C2	lepB	-2.3	-2.7	0.10
C3	fimI	-5.5	-5.7	0.68
	lsrR	-2.2	-2.4	0.68
C4	ydcS	-2.9	-3.2	0.53
	dksA	-1.9	-2.0	0.51
	pdeD	-4.0	-4.2	0.39
	rfaD	-1.0	-1.0	0.79
	kdsB	-3.7	-3.5	0.59
C5	lpxH	-5.5	-5.8	0.25
	ftsI	-4.5	-4.6	0.90
	waaH	-6.0	-6.2	0.53

Cluster	Gene	T	L	p-value
	hokC	-3.0	-3.3	0.27
	fadL	-1.9	-2.1	0.69
	adeQ	-7.5	-8.7	0.20
	exuT	-12.6	-12.1	0.09
	sdaC	-4.6	-4.5	0.71
	ppdA	-6.7	-6.6	0.87
	aqpZ	-3.4	-3.6	0.55
	relE	-1.8	-1.7	0.70
	nhaA	-2.7	-3.0	0.33
	hokD	-1.7	-1.6	0.73
	yahN	-6.8	-7.4	0.08
	cycA	-1.8	-1.6	0.70
	bssS	2.3	1.9	0.03
	hokB	-5.3	-4.9	0.14
	eamB	-5.9	-6.3	0.19
	bssR	2.1	2.4	0.35
	kdgT	-6.2	-6.4	0.50
	crcB	-3.7	-3.7	0.91
	ygbN	-7.3	-7.6	0.60
K6	cirA	-7.8	-7.9	0.46
	aroP	-3.1	-3.4	0.67
	ydhP	-3.9	-3.7	0.45
	lptD	-2.2	-2.1	0.83
	yccA	1.0	1.0	0.95
	tomB	0.5	0.7	0.48
	xylE	-7.9	-8.6	0.12
	puuP	-4.8	-4.8	1.00
	zitB	-3.9	-4.0	0.95
	mdtE	-2.8	-2.3	0.44
	blc	-1.5	-1.7	0.41
	mscS	-1.6	-1.3	0.42
	amtB	-3.9	-2.6	0.08
	ycaD	-2.7	-2.3	0.44
	ybhG	-2.7	-2.5	0.76
	galP	-5.2	-5.1	0.85
	ydeA	-5.8	-5.2	0.09
	kefF	-5.8	-5.3	0.26
	phoE	-8.7	-9.1	0.02
	csgE	-4.1	-4.2	0.50

Fig. S4. Quantification of mRNA levels of selected differentially expressed genes (DEGs) using quantitative reverse transcription polymerase chain reaction (RT-qPCR). Relative mRNA levels were calculated under Tris buffer (15 min) and LNT113 (0.64 µM, 15 min) conditions. Ct values for each gene were normalized to *rpoD*, and relative expression levels are presented as *Δ*Ct values in T (Tris buffer 15 min) and L (LNT113 15 min) columns. Yellow-highlighted genes exhibited statistically significant differences between two conditions ($p < 0.05$).

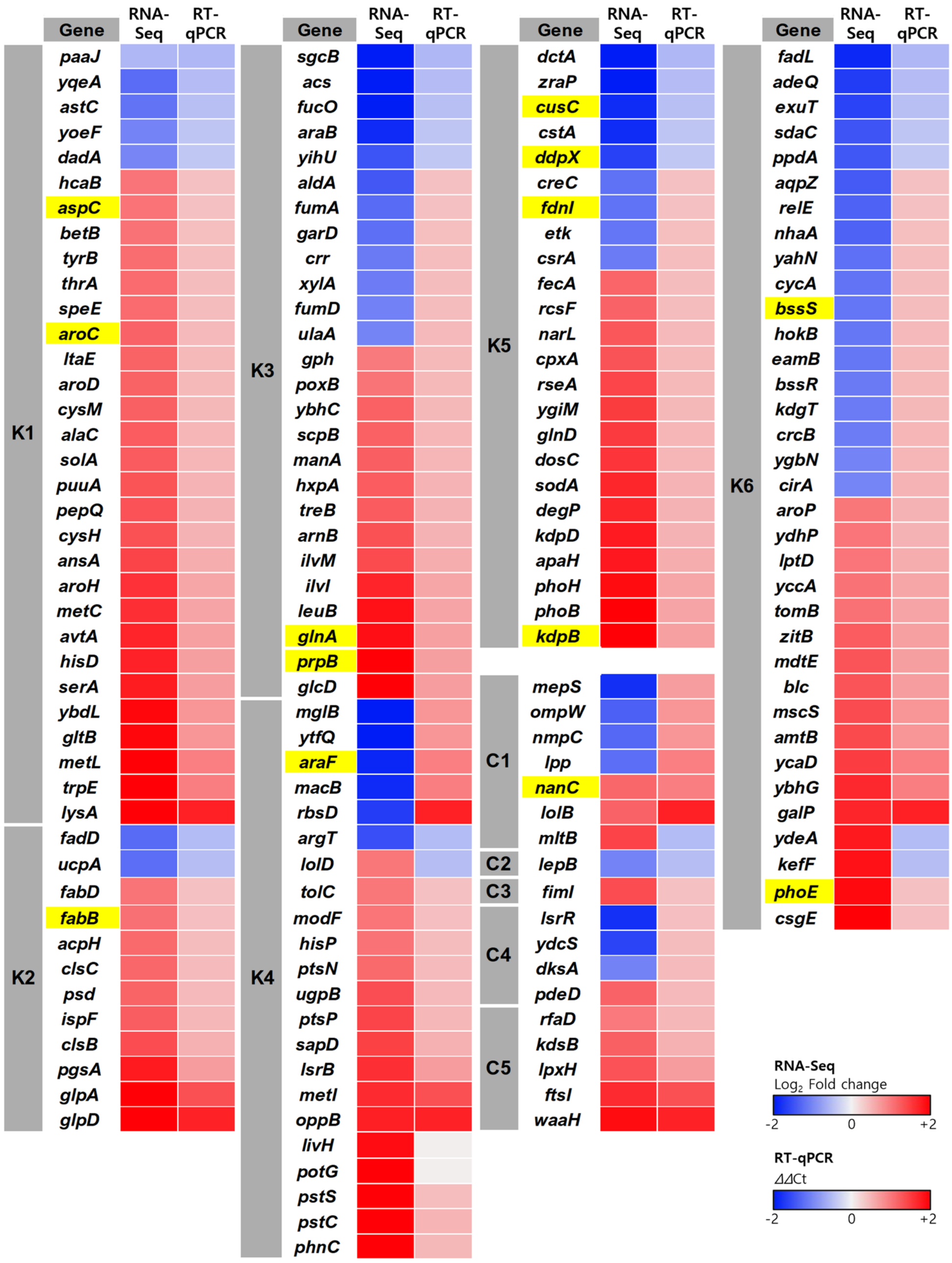

Fig. S5. Comparison of expression patterns between RNA-seq and RT-qPCR. Heat maps illustrate the expression profiles of 168 differentially expressed genes (DEGs) determined by RNA-seq (left column of each pair) and RT-qPCR (right column of each pair). Genes are organized according to their KEGG and COG functional categories, with each row corresponding to a single gene. Expression changes induced by LNT113 treatment are represented by color intensity, where red denotes upregulated genes and blue denotes downregulated genes. RT-qPCR expression levels are reported as *ΔΔ*Ct values, calculated by subtracting the *Δ*Ct of the control group (Tris buffer, 15 min) from that of the LNT113-treated group (15 min), with *Δ*Ct defined as $Ct_{[rpoD]} - Ct_{[\text{target gene}]}$ (n = 2 per group). RNA-seq results are shown as fold changes between the LNT113-treated (15 min) and control (Tris buffer, 15 min) samples. Genes highlighted in yellow exhibited statistically significant differences in RT-qPCR analysis ($p < 0.05$) and were subsequently selected for functional validation.

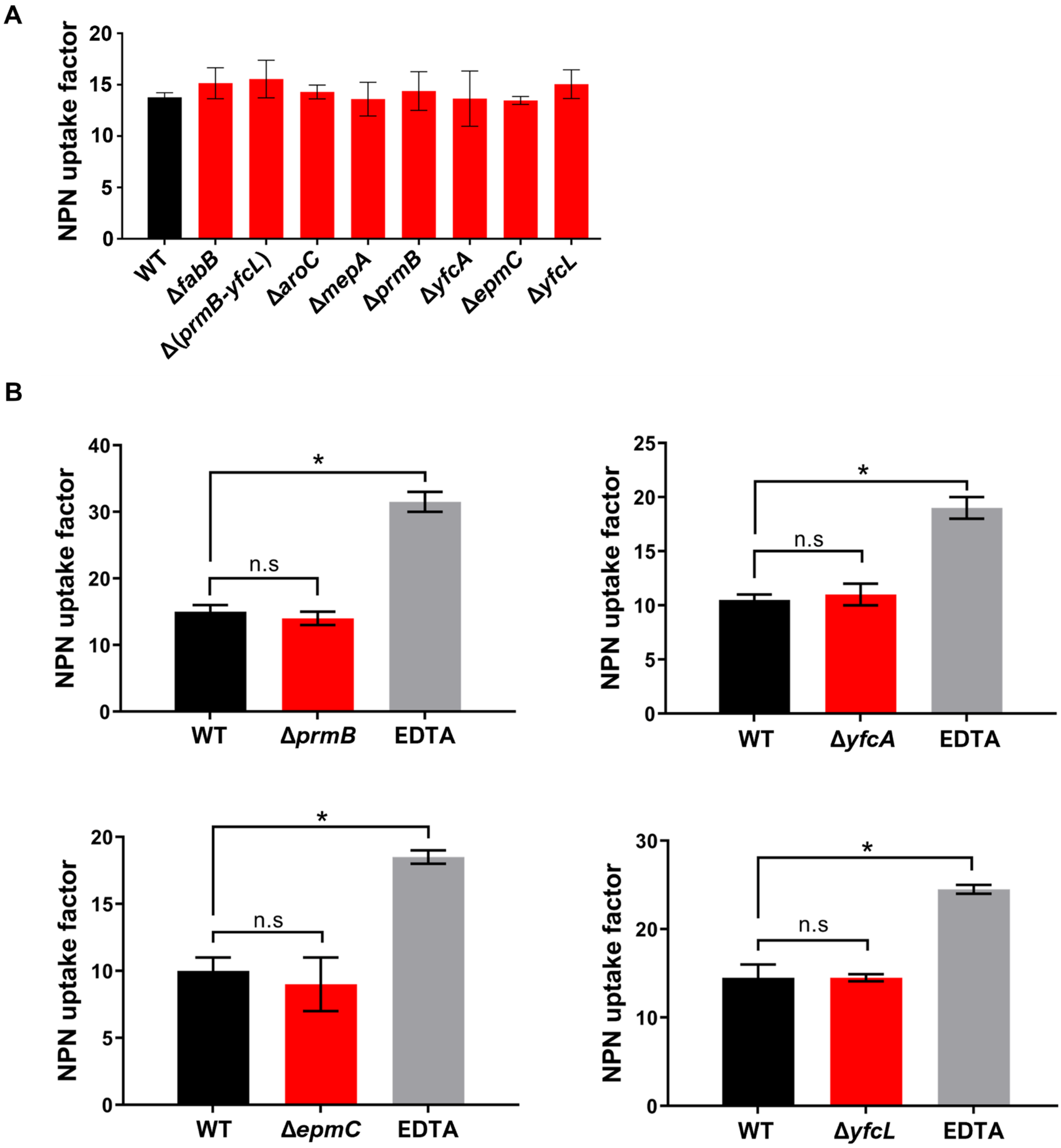

Fig. S6. Comparison of NPN uptake in bacteria lacking candidate genes. (A) Membrane permeability was assessed by the NPN uptake assay in *Escherichia coli* MG1655 wild-type, Δ*fabB*, Δ(*prmB*–*yfcL*), Δ*aroC*, Δ*mepA*, Δ*prmB*, Δ*yfcA*, Δ*epmC*, and Δ*yfcL* strains in the absence of LNT113 treatment. (B) Bacterial membrane permeability was assessed in Δ*prmB*, Δ*yfcA*, Δ*epmC*, and Δ*yfcL*. Bacterial cells were treated with 0.64 μM LNT113 for 5 min. Negative control: wild-type *Escherichia coli* MG1655; positive control: 1 mM EDTA-treated *E. coli* MG1655 cells. Data represent the Mean ± standard error of the mean from three biological replicates. Statistically significant differences ($p < 0.05$) are denoted by asterisks.

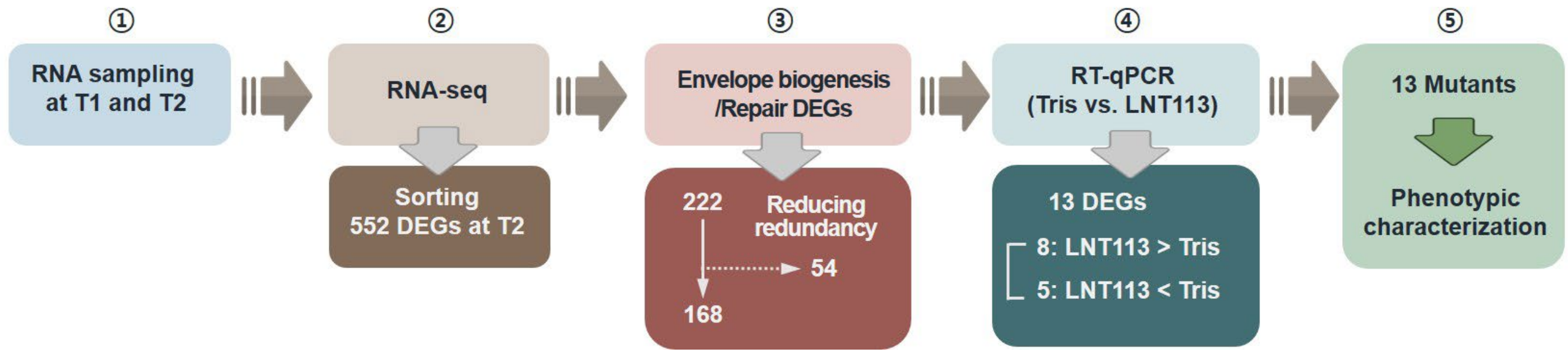

Fig. S7. Schematic analytical flow. ① Comparative transcriptomic analysis was conducted under T1 (Tris buffer, 15 min vs. Tris buffer, 0 min) and T2 (LNT113, 15 min vs. Tris buffer, 15 min) conditions. ② A total of 552 DEGs were selected from T2 condition and ③ then narrowed down to 168 DEGs, prioritizing those associated with cell damage repair and metabolic adaptation to stress. ④ The set of 168 DEGs were subjected to RT-qPCR under Tris buffer (15 min) and LNT113 (15 min) conditions and thirteen DEGs with different transcription levels between two conditions ($p < 0.05$) were finally chosen as candidate resistance determinants against endolysin LNT113. ⑤ Bacterial mutant strains lacking candidate DEGs were constructed and their phenotypic characteristics were investigated to determine the genes critical for bacterial tolerance to LNT113.
