# Supplementary material for: Transcriptomic insights into the effects of sublethal exposure to endolysin LNT113 on Escherichia coli
Source: J Microbiol. 2026 Jul 31;64(7):e2605009. doi: 10.71150/jm.2605009 (PMC13714653; doi:10.71150/jm.2605009)
Supplement: Supplementary file 2 [file jm-2605009-Supplementary-Fig-S2.pdf]

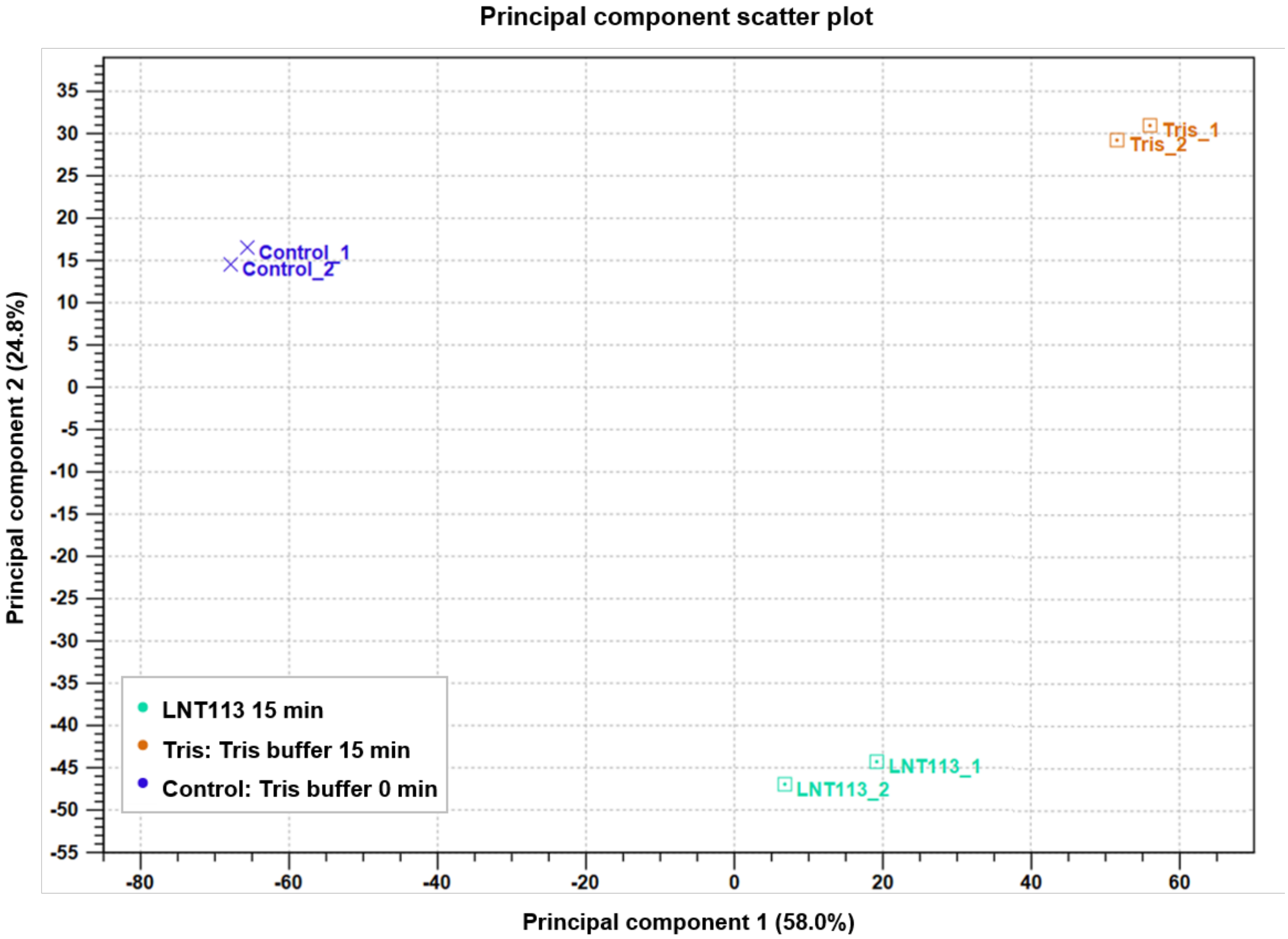


**Fig. S2.** Principal component analysis (PCA) of RNA sequencing results. PCA showed tight clustering of biological replicates, depending on the RNA preparation conditions. Samples from the Tris buffer 15 min and LNT113 15 min were clearly separated from those of the Tris buffer 0 min control, indicating high reproducibility and treatment-dependent transcriptional differences.
