# Supplementary material for: Transcriptomic insights into the effects of sublethal exposure to endolysin LNT113 on Escherichia coli
Source: J Microbiol. 2026 Jul 31;64(7):e2605009. doi: 10.71150/jm.2605009 (PMC13714653; doi:10.71150/jm.2605009)
Supplement: Supplementary file 3 [file jm-2605009-Supplementary-Fig-S3.pdf]

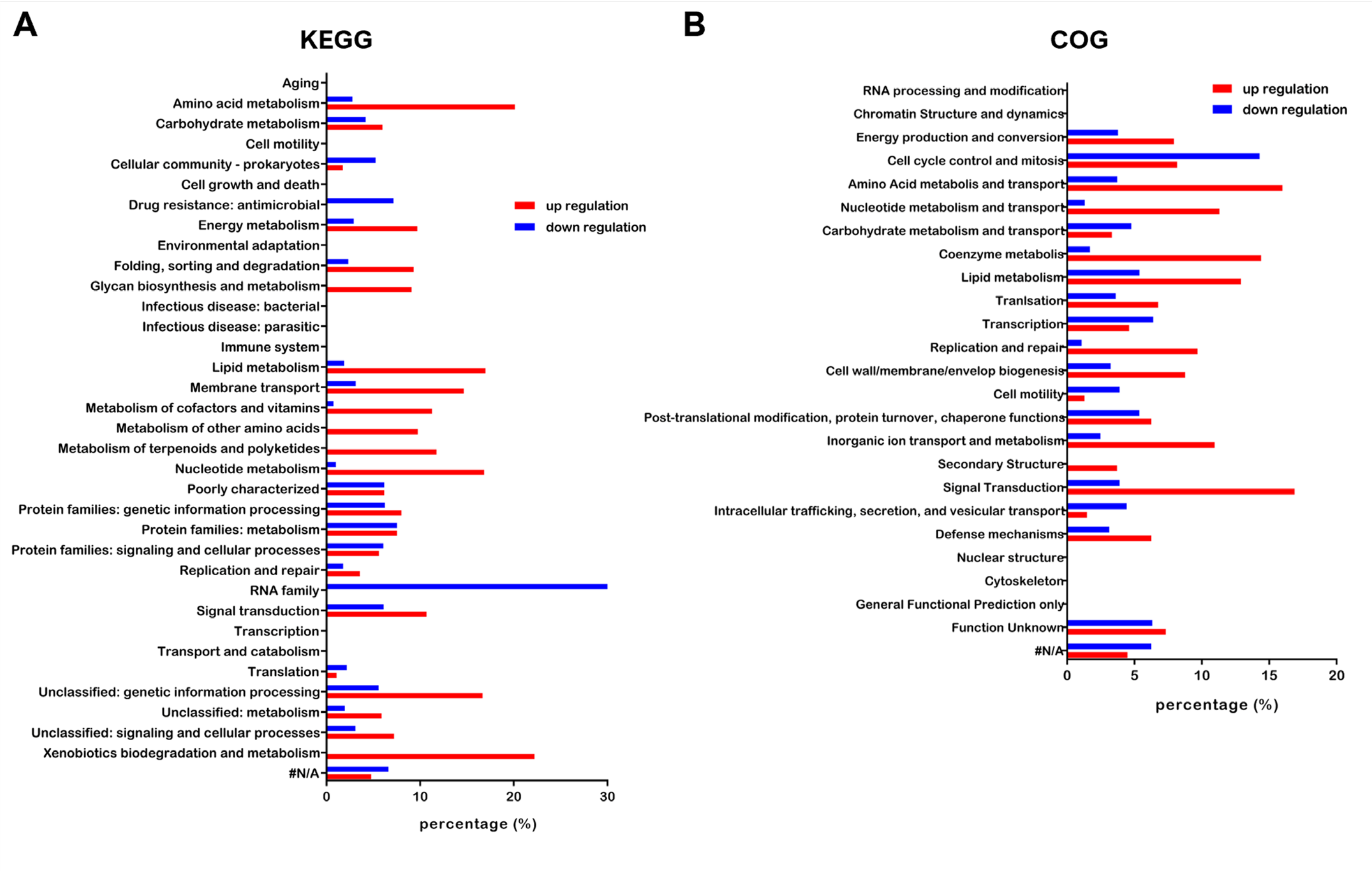

Fig. S3. Functional categorization of genes with altered mRNA levels following LNT113 treatment. Differentially expressed genes (DEGs) were grouped based on (A) Kyoto Encyclopedia of Genes and Genomes (KEGG) classification and (B) Clusters of Orthologous Groups (COG) analysis. Red and blue bars represent the percentages of genes with upregulated and downregulated expressions, respectively, in each functional category.
