# Supplementary material for: Transcriptomic insights into the effects of sublethal exposure to endolysin LNT113 on Escherichia coli
Source: J Microbiol. 2026 Jul 31;64(7):e2605009. doi: 10.71150/jm.2605009 (PMC13714653; doi:10.71150/jm.2605009)
Supplement: Supplementary file 4 [file jm-2605009-Supplementary-Fig-S4.pdf]

Group	Gene	T	L	p-value
K1	*paaJ*	-6.0	-5.6	0.32
K1	*paaK*	-3.8	-3.1	0.35
K1	*yqeA*	-5.4	-5.1	0.37
K1	*astC*	-1.0	-0.8	0.62
K1	*yoeF*	-5.8	-5.5	0.42
K1	*dadA*	0.5	0.2	0.59
K1	*hcaB*	-4.9	-5.2	0.13
K1	*aspC*	-1.3	-1.0	0.03
K1	*betB*	-3.7	-3.5	0.67
K1	*tyrB*	-2.0	-2.2	0.48
K1	*thrA*	-2.8	-2.3	0.21
K1	*speE*	-1.3	-1.2	0.81
K1	*aroC*	-3.5	-2.8	0.04
K1	*ltaE*	-1.2	-1.0	0.84
K1	*aroD*	-3.1	-3.1	0.89
K1	*cysM*	-2.1	-2.0	0.62
K1	*alaC*	-3.5	-3.6	0.43
K1	*solA*	-5.1	-4.8	0.21
K1	*puuA*	-4.5	-3.2	0.06
K1	*pepQ*	-2.1	-1.8	0.11
K1	*cysH*	-1.4	-1.1	0.15
K1	*metA*	-1.5	-0.9	0.34
K1	*ansA*	-3.4	-3.9	0.29
K1	*aroH*	-2.3	-2.3	0.98
K1	*metC*	-4.1	-3.8	0.66
K1	*avtA*	-2.7	-2.5	0.82
K1	*hisD*	-1.0	-0.5	0.27
K1	*serA*	-0.7	0.0	0.08
K1	*ybdL*	-2.4	-1.8	0.48
K1	*gltB*	-0.4	-0.2	0.64
K1	*metL*	-2.2	-1.9	0.28
K1	*trpE*	1.8	2.2	0.18
K1	*hisB*	-2.0	-2.0	0.84
K1	*lysA*	-7.8	-5.9	0.06
K1	*trpB*	0.3	0.2	0.89
K2	*fadD*	0.9	0.3	0.08
K2	*ucpA*	-1.9	-2.0	0.81
K2	*fabD*	-0.5	-0.5	0.93
K2	*fabB*	-1.4	-0.8	0.04
K2	*acpH*	-4.5	-4.4	0.29
K2	*clsC*	-3.5	-3.3	0.51
K2	*psd*	-3.3	-2.8	0.19
K2	*ispF*	-3.8	-3.6	0.59
K2	*clsB*	-4.7	-4.6	0.87
K2	*pgsA*	-4.2	-3.8	0.23
K2	*glpA*	-8.2	-6.6	0.36
K2	*glpD*	-3.8	-2.6	0.18

Group	Gene	T	L	p-value
K3	*sgcB*	-5.2	-5.1	0.66
K3	*yqeF*	-3.1	-2.8	0.26
K3	*acs*	-0.2	-0.2	0.95
K3	*fucO*	-4.1	-3.8	0.36
K3	*araB*	-8.4	-8.3	0.56
K3	*yihU*	-8.7	-8.7	0.99
K3	*aldA*	0.7	0.6	0.85
K3	*fumA*	-0.6	-0.6	0.94
K3	*garD*	-5.7	-5.7	0.88
K3	*crr*	1.5	1.8	0.35
K3	*xylA*	-6.1	-4.5	0.08
K3	*fumD*	-4.8	-4.3	0.20
K3	*ulaA*	-7.9	-7.5	0.30
K3	*gph*	-3.3	-3.1	0.24
K3	*poxB*	-1.5	-1.2	0.06
K3	*ybhC*	-5.1	-4.8	0.51
K3	*scpB*	-7.7	-7.4	0.66
K3	*manA*	-1.7	-1.7	0.89
K3	*hxpA*	-4.0	-3.7	0.39
K3	*treB*	-6.4	-6.2	0.50
K3	*arnB*	-5.1	-4.9	0.47
K3	*ilvM*	-4.8	-4.6	0.44
K3	*ilvI*	-5.5	-5.4	0.79
K3	*treF*	-2.0	-1.9	0.39
K3	*leuB*	-2.9	-2.8	0.77
K3	*glnA*	0.5	1.7	0.05
K3	*prpB*	-2.1	-1.0	0.02
K3	*glcD*	-1.4	-1.2	0.41
K4	*mglB*	-2.8	-3.4	0.17
K4	*ytfQ*	-2.6	-2.6	0.84
K4	*araF*	-5.5	-4.9	0.01
K4	*macB*	-5.1	-4.9	0.25
K4	*rbsD*	0.5	0.1	0.16
K4	*argT*	-1.2	-1.3	0.83
K4	*lolD*	-3.5	-3.2	0.32
K4	*tolC*	-1.7	-1.2	0.13
K4	*modF*	-5.9	-5.6	0.30
K4	*hisP*	-6.1	-5.9	0.84
K4	*potB*	-4.3	-4.0	0.26
K4	*ptsN*	-1.7	-1.4	0.47
K4	*ugpB*	-3.8	-3.8	0.97
K4	*ptsP*	-3.0	-2.8	0.31
K4	*sapD*	-2.9	-2.8	0.36
K4	*lsrB*	-2.9	-2.3	0.24
K4	*metI*	-3.3	-3.4	0.94
K4	*oppB*	-0.3	0.5	0.31
K4	*livH*	-7.2	-6.8	0.38
K4	*livJ*	-4.9	-4.8	0.30
K4	*potG*	-5.3	-5.3	0.95
K4	*pstS*	-1.9	-1.7	0.76
K4	*pstC*	-3.9	-3.8	0.93
K4	*pstB*	-3.3	-3.5	0.85
K4	*phnC*	-9.6	-9.1	0.36

Group	Gene	T	L	p-value
K3	*sgcB*	-5.2	-5.1	0.66
K3	*yqeF*	-3.1	-2.8	0.26
K3	*acs*	-0.2	-0.2	0.95
K3	*fucO*	-4.1	-3.8	0.36
K3	*araB*	-8.4	-8.3	0.56
K3	*yihU*	-8.7	-8.7	0.99
K3	*aldA*	0.7	0.6	0.85
K3	*fumA*	-0.6	-0.6	0.94
K3	*garD*	-5.7	-5.7	0.88
K3	*crr*	1.5	1.8	0.35
K3	*xylA*	-6.1	-4.5	0.08
K3	*fumD*	-4.8	-4.3	0.20
K3	*ulaA*	-7.9	-7.5	0.30
K3	*gph*	-3.3	-3.1	0.24
K3	*poxB*	-1.5	-1.2	0.06
K3	*ybhC*	-5.1	-4.8	0.51
K3	*scpB*	-7.7	-7.4	0.66
K3	*manA*	-1.7	-1.7	0.89
K3	*hxpA*	-4.0	-3.7	0.39
K3	*treB*	-6.4	-6.2	0.50
K3	*arnB*	-5.1	-4.9	0.47
K3	*ilvM*	-4.8	-4.6	0.44
K3	*ilvI*	-5.5	-5.4	0.79
K3	*treF*	-2.0	-1.9	0.39
K3	*leuB*	-2.9	-2.8	0.77
K3	*glnA*	0.5	1.7	0.05
K3	*prpB*	-2.1	-1.0	0.02
K3	*glcD*	-1.4	-1.2	0.41
K4	*mglB*	-2.8	-3.4	0.17
K4	*ytfQ*	-2.6	-2.6	0.84
K4	*araF*	-5.5	-4.9	0.01
K4	*macB*	-5.1	-4.9	0.25
K4	*rbsD*	0.5	0.1	0.16
K4	*argT*	-1.2	-1.3	0.83
K4	*lolD*	-3.5	-3.2	0.32
K4	*tolC*	-1.7	-1.2	0.13
K4	*modF*	-5.9	-5.6	0.30
K4	*hisP*	-6.1	-5.9	0.84
K4	*potB*	-4.3	-4.0	0.26
K4	*ptsN*	-1.7	-1.4	0.47
K4	*ugpB*	-3.8	-3.8	0.97
K4	*ptsP*	-3.0	-2.8	0.31
K4	*sapD*	-2.9	-2.8	0.36
K4	*lsrB*	-2.9	-2.3	0.24
K4	*metI*	-3.3	-3.4	0.94
K4	*oppB*	-0.3	0.5	0.31
K4	*livH*	-7.2	-6.8	0.38
K4	*livJ*	-4.9	-4.8	0.30
K4	*potG*	-5.3	-5.3	0.95
K4	*pstS*	-1.9	-1.7	0.76
K4	*pstC*	-3.9	-3.8	0.93
K4	*pstB*	-3.3	-3.5	0.85
K4	*phnC*	-9.6	-9.1	0.36

Group	Gene	T	L	p-value
K6	*hokC*	-3.0	-3.3	0.27
K6	*fadL*	-1.9	-2.1	0.69
K6	*adeQ*	-7.5	-8.7	0.20
K6	*exuT*	-12.6	-12.1	0.09
K6	*sdaC*	-4.6	-4.5	0.71
K6	*ppdA*	-6.7	-6.6	0.87
K6	*aqpZ*	-3.4	-3.6	0.55
K6	*relE*	-1.8	-1.7	0.70
K6	*nhaA*	-2.7	-3.0	0.33
K6	*hokD*	-1.7	-1.6	0.73
K6	*yahN*	-6.8	-7.4	0.08
K6	*cycA*	-1.8	-1.6	0.70
K6	*bssS*	2.3	1.9	0.03
K6	*hokB*	-5.3	-4.9	0.14
K6	*eamB*	-5.9	-6.3	0.19
K6	*bssR*	2.1	2.4	0.35
K6	*kdgT*	-6.2	-6.4	0.50
K6	*crcB*	-3.7	-3.7	0.91
K6	*ygbN*	-7.3	-7.6	0.60
K6	*cirA*	-7.8	-7.9	0.46
K6	*aroP*	-3.1	-3.4	0.67
K6	*ydhP*	-3.9	-3.7	0.45
K6	*lptD*	-2.2	-2.1	0.83
K6	*yccA*	1.0	1.0	0.95
K6	*tomB*	0.5	0.7	0.48
K6	*xylE*	-7.9	-8.6	0.12
K6	*puuP*	-4.8	-4.8	1.00
K6	*zitB*	-3.9	-4.0	0.95
K6	*mdtE*	-2.8	-2.3	0.44
K6	*blc*	-1.5	-1.7	0.41
K6	*mscS*	-1.6	-1.3	0.42
K6	*amtB*	-3.9	-2.6	0.08
K6	*ycaD*	-2.7	-2.3	0.44
K6	*ybhG*	-2.7	-2.5	0.76
K6	*galP*	-5.2	-5.1	0.85
K6	*ydeA*	-5.8	-5.2	0.09
K6	*kefF*	-5.8	-5.3	0.26
K6	*phoE*	-8.7	-9.1	0.02
K6	*csgE*	-4.1	-4.2	0.50

Fig. S4. Quantification of mRNA levels of selected differentially expressed genes (DEGs) using quantitative reverse transcription polymerase chain reaction (RT-qPCR). Relative mRNA levels were calculated under Tris buffer (15 min) and LNT113 (0.64 µM, 15 min) conditions. Ct values for each gene were normalized to *rpoD*, and relative expression levels are presented as *Δ*Ct values in T (Tris buffer 15 min) and L (LNT113 15 min) columns. Yellow-highlighted genes exhibited statistically significant differences between two conditions ($p < 0.05$).
