# Supplementary material for: Transcriptomic insights into the effects of sublethal exposure to endolysin LNT113 on Escherichia coli
Source: J Microbiol. 2026 Jul 31;64(7):e2605009. doi: 10.71150/jm.2605009 (PMC13714653; doi:10.71150/jm.2605009)
Supplement: Supplementary file 5 [file jm-2605009-Supplementary-Fig-S5.pdf]

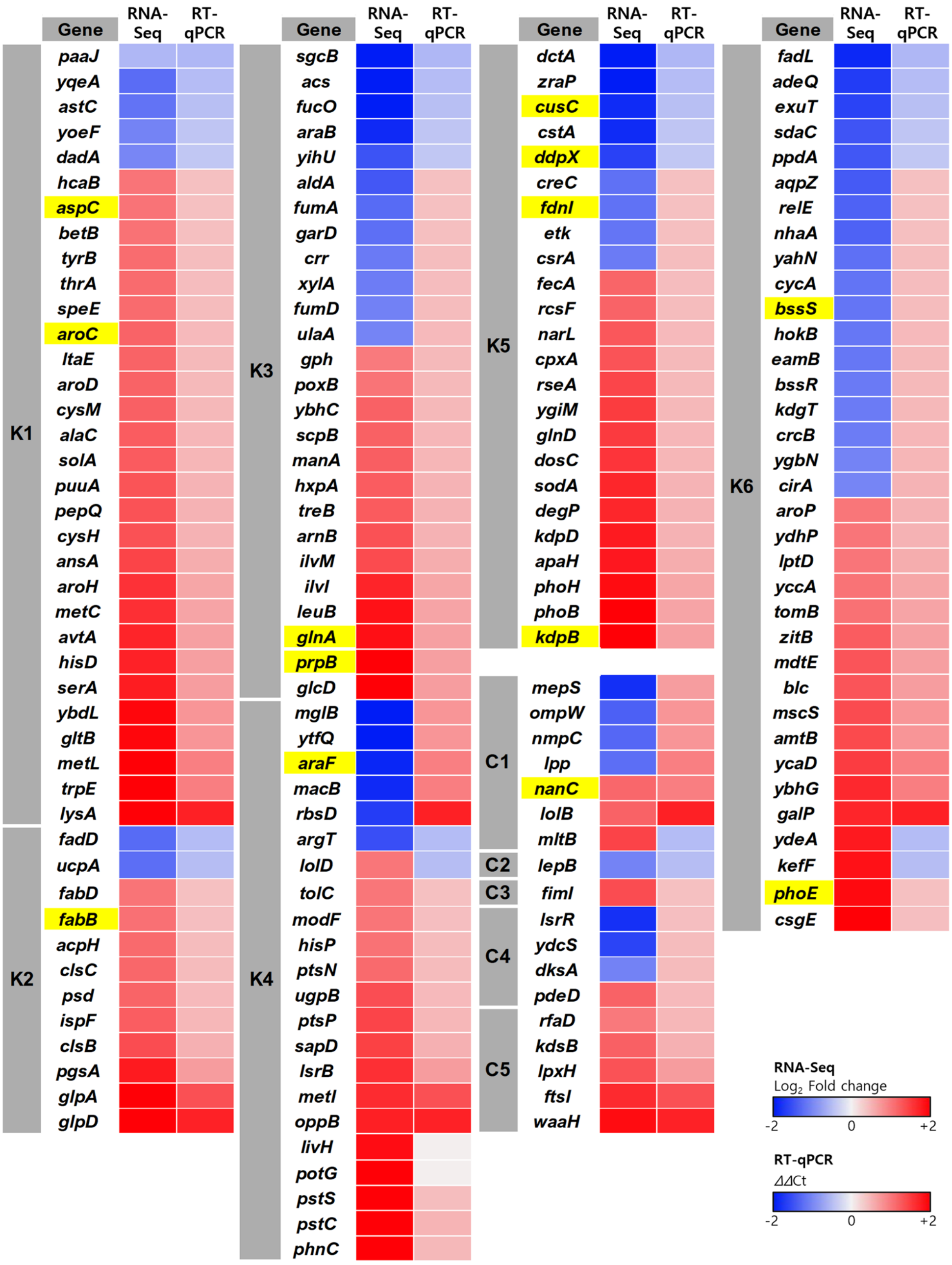

Fig. S5. Comparison of expression patterns between RNA-seq and RT-qPCR. Heat maps illustrate the expression profiles of 168 differentially expressed genes (DEGs) determined by RNA-seq (left column of each pair) and RT-qPCR (right column of each pair). Genes are organized according to their KEGG and COG functional categories, with each row corresponding to a single gene. Expression changes induced by LNT113 treatment are represented by color intensity, where red denotes upregulated genes and blue denotes downregulated genes. RT-qPCR expression levels are reported as *ΔΔ*Ct values, calculated by subtracting the *Δ*Ct of the control group (Tris buffer, 15 min) from that of the LNT113-treated group (15 min), with *Δ*Ct defined as $Ct_{[rpoD]} - Ct_{[target\ gene]}$ (n = 2 per group). RNA-seq results are shown as fold changes between the LNT113-treated (15 min) and control (Tris buffer, 15 min) samples. Genes highlighted in yellow exhibited statistically significant differences in RT-qPCR analysis ($p < 0.05$) and were subsequently selected for functional validation.
