# Supplementary material for: Transcriptomic insights into the effects of sublethal exposure to endolysin LNT113 on Escherichia coli
Source: J Microbiol. 2026 Jul 31;64(7):e2605009. doi: 10.71150/jm.2605009 (PMC13714653; doi:10.71150/jm.2605009)
Supplement: Supplementary file 6 [file jm-2605009-Supplementary-Fig-S6.pdf]

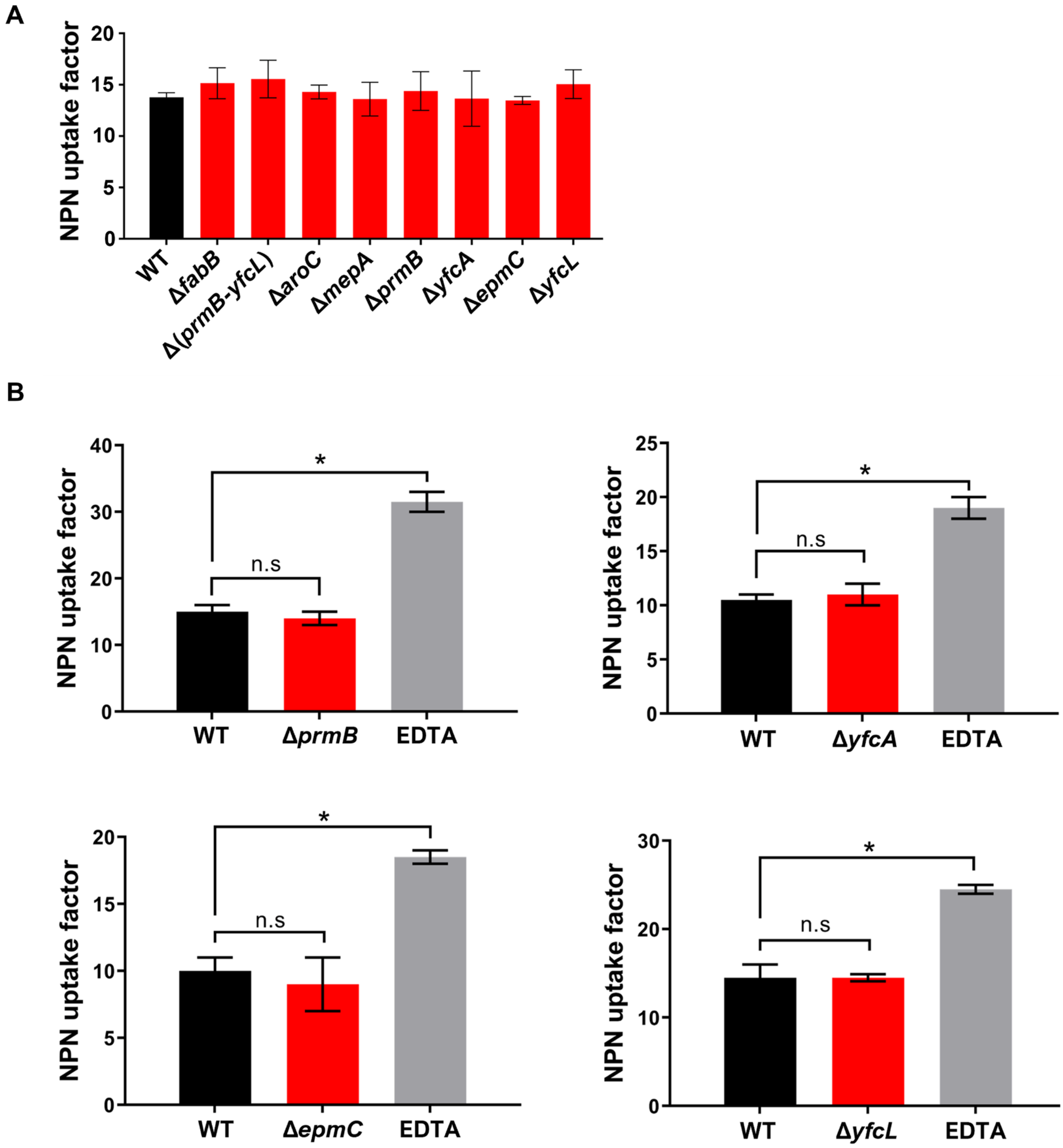

Fig. S6. Comparison of NPN uptake in bacteria lacking candidate genes. (A) Membrane permeability was assessed by the NPN uptake assay in *Escherichia coli* MG1655 wild-type, Δ*fabB*, Δ(*prmB*–*yfcL*), Δ*aroC*, Δ*mepA*, Δ*prmB*, Δ*yfcA*, Δ*epmC*, and Δ*yfcL* strains in the absence of LNT113 treatment. (B) Bacterial membrane permeability was assessed in Δ*prmB*, Δ*yfcA*, Δ*epmC*, and Δ*yfcL*. Bacterial cells were treated with 0.64 μM LNT113 for 5 min. Negative control: wild-type *Escherichia coli* MG1655; positive control: 1 mM EDTA-treated *E. coli* MG1655 cells. Data represent the Mean ± standard error of the mean from three biological replicates. Statistically significant differences ($p < 0.05$) are denoted by asterisks.
