# Supplementary material for: Transcriptomic insights into the effects of sublethal exposure to endolysin LNT113 on Escherichia coli
Source: J Microbiol. 2026 Jul 31;64(7):e2605009. doi: 10.71150/jm.2605009 (PMC13714653; doi:10.71150/jm.2605009)
Supplement: Supplementary file 7 [file jm-2605009-Supplementary-Fig-S7.pdf]

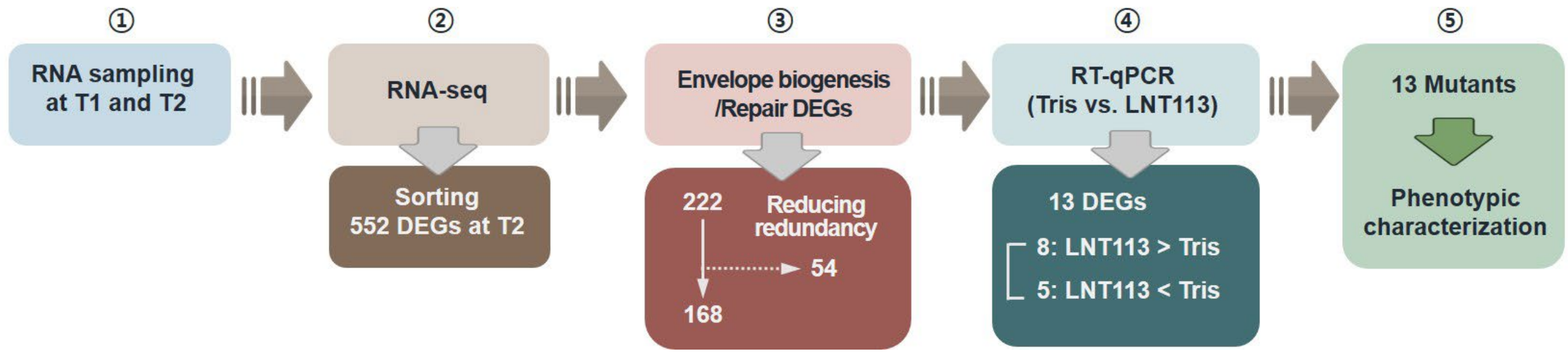

Fig. S7. Schematic analytical flow. ① Comparative transcriptomic analysis was conducted under T1 (Tris buffer, 15 min vs. Tris buffer, 0 min) and T2 (LNT113, 15 min vs. Tris buffer, 15 min) conditions. ② A total of 552 DEGs were selected from T2 condition and ③ then narrowed down to 168 DEGs, prioritizing those associated with cell damage repair and metabolic adaptation to stress. ④ The set of 168 DEGs were subjected to RT-qPCR under Tris buffer (15 min) and LNT113 (15 min) conditions and thirteen DEGs with different transcription levels between two conditions ($p < 0.05$) were finally chosen as candidate resistance determinants against endolysin LNT113. ⑤ Bacterial mutant strains lacking candidate DEGs were constructed and their phenotypic characteristics were investigated to determine the genes critical for bacterial tolerance to LNT113.
