# Supplementary material for: Transcriptomic insights into the effects of sublethal exposure to endolysin LNT113 on Escherichia coli
Source: J Microbiol. 2026 Jul 31;64(7):e2605009. doi: 10.71150/jm.2605009 (PMC13714653; doi:10.71150/jm.2605009)
Supplement: Supplementary file 8 [file jm-2605009-Supplementary-Table-S1.pdf]

Table S1. Bacterial strains used in this study

Bacterial strain	Source
Escherichia coli K-12 substr. MG1655 (GenBank:U00096.3)	ATCC[a] 700926
E. coli BL21(DE3) star: F^- *ompT hsdSB*(*rB*$^-$ *mB*$^-$) *gal dcm rne131* λ(DE3)Δ*lacZ*	ATCC BAA-1025-B2
E. coli MG1655, Δ*aspC*	This study
E. coli MG1655, Δ(*prmB-yfcL*)	This study
E. coli MG1655, Δ *fabB*	This study
E. coli MG1655, Δ*glnALG*	This study
E. coli MG1655, Δ*prpBCDE*	This study
E. coli MG1655, Δ*araFGH*	This study
E. coli MG1655, Δ*cusCFBA*	This study
E. coli MG1655, Δ*ddpXABCDF*	This study
E. coli MG1655, Δ*fdnGHI*	This study
E. coli MG1655, Δ*kdpFABC*	This study
E. coli MG1655, Δ*bssS*	This study
E. coli MG1655, Δ*phoE*	This study
E. coli MG1655, Δ*nanCMS*	This study
E. coli MG1655, Δ*aroC*	This study
E. coli MG1655, Δ*mepA*	This study
E. coli MG1655, Δ*prmB*	This study
E. coli MG1655, Δ*yfcA*	This study
E. coli MG1655, Δ*epmC*	This study
E. coli MG1655, Δ*yfcL*	This study

[a]ATCC, American Type Culture Collection, USA.
