# Supplementary material for: Transcriptomic insights into the effects of sublethal exposure to endolysin LNT113 on Escherichia coli
Source: J Microbiol. 2026 Jul 31;64(7):e2605009. doi: 10.71150/jm.2605009 (PMC13714653; doi:10.71150/jm.2605009)
Supplement: Supplementary file 9 [file jm-2605009-Supplementary-Table-S2.pdf]

Table S2. Primers used in strain construction

Primer name	Sequence (from 5' to 3')
*aspC*_RF	ATAGCGGACTTCCCTTCTGTAACCATAATGGAACCTCGTCATTCCGGGGATCCGTCGACC
*aspC*_RR	TCTCAGCCCGCTTTTCAGCGGGCTTCATTGTTTTTAATGCGTGTAGGCTGGAGCTGCTTC
(*prmB-yfcL*)_RF	CGGTGGCTTACCTGACCAACAAAGCGTGGTTCTGCGGCCAATTCCGGGGATCCGTCGACC
(*prmB-yfcL*)_RR	ATAAGCGTCGCATCAGGCATGCTGCTCCAGACGCCGCAACGTGTAGGCTGGAGCTGCTTC
*glnALG*_RF	CACCGATACTAAAGGTAAAGAACAGCACGTCACTATCCCTATTCCGGGGATCCGTCGACC
*glnALG*_RR	ATTTGCGCTCAATAATCAATCTTTACACACAAGCTGTGAAGTGTAGGCTGGAGCTGCTTC
*fabB*_RF	ATTCGAAACTTACTCTATGTGCGACTTACAGAGGTATTGAATTCCGGGGATCCGTCGACC
*fabB*_RR	CAGCCTGCTCGTCCAGCTCTTCAATGTTGATGCTCGGGGCGTGTAGGCTGGAGCTGCTTC
*araFGH*_RF	TTTTGCCCTACACAAAACGACACTAAAGCTGGAGAGAACCATTCCGGGGATCCGTCGACC
*araFGH*_RR	CCACCTTTCAGAGAAACGCCACCTAAAACGCAGGCGGAGAGTGTAGGCTGGAGCTGCTTC
*bssS*_RF	TCGAATAACGTTGTCTAGTAACACGAATTAGGGGGCCATGATTCCGGGGATCCGTCGACC
*bssS*_RR	TTTACCTGGAAATCACCGGATTCAATTTTGGCGATTCCTGGTGTAGGCTGGAGCTGCTTC
*phoE*_RF	ATCTGCATCTGTACAGGCTGCAGAAATATATAATAAAGACATTCCGGGGATCCGTCGACC
*phoE*_RR	GAGTGAAATGTGCCGGATGCATCACATCCGGCAATATTCAGTGTAGGCTGGAGCTGCTTC
*cusCFBA*_RF	CTGGTTAACGCAGCAGATAACTATCAGAACGCGGGCTGGCATTCCGGGGATCCGTCGACC
*cusCFBA*_RR	CGCTTTCGGGCGCACGCGCAGGACCGCGCCGTGATATAACGTGTAGGCTGGAGCTGCTTC
*ddpXABCDF*_RF	CGGAAAGCGCAGCAATTTTTGTCTTATACAGGAACCGTTTATTCCGGGGATCCGTCGACC
*ddpXABCDF*_RR	CGCGTCAGGCGCGTGAATGCCTGATGCGTTGTTAGCATCTGTGTAGGCTGGAGCTGCTTC
*fdnGHI*_RF	GGCATTGGGCTTTGCCCCGAAGCAAGCACTGGCTCAGGCGATTCCGGGGATCCGTCGACC
*fdnGHI*_RR	CCTTTTACAGCGCCATTTTCAGCTTAAAGTTCAAAAGAGAGTGTAGGCTGGAGCTGCTTC
*prpBCDE*_RF	TTATCAACGCAAATAACAAGTTGATAACAAAGGATGGGCTATTCCGGGGATCCGTCGACC
*prpBCDE*_RR	CACAGTGCCGGATGCACCGCGCGACGCATCCGACAATAACGTGTAGGCTGGAGCTGCTTC
*nanCMS*_RF	CTTTGTATTGCTTTCTGTATCCTACAAATGAGTGAAATTTATTCCGGGGATCCGTCGACC
*nanCMS*_RR	GTCAGAAATAATCCCCCGACGGGCTGCAGTGCTGAAATGAGTGTAGGCTGGAGCTGCTTC
*kdpFABC*_RF	TACCCTTCCGGTATCACTTTTAGGCCACTGGAGGTGCACTATTCCGGGGATCCGTCGACC
*kdpFABC*_RR	GCAGACGATCGGGGTCGGGACGTAAGGGTTCGTTATTCATGTGTAGGCTGGAGCTGCTTC
*prmB*_RF	TGCCTTCGCTCTACCTGCCGCTGGATATTCCGGAAGATATATTCCGGGGATCCGTCGACC
*prmB*_RR	AGCCATCACGGCTCCGTTATTGTTGTGTTTGCGTGTTTACGTGTAGGCTGGAGCTGCTTC
*aroC*_RF	GATTAAGTAAACACGCAAACACAACAATAACGGAGCCGTGATTCCGGGGATCCGTCGACC
*aroC*_RR	CAAGCAGAGCCAGCAGCGCAATCGCGGTTTTATTCATTTTGTGTAGGCTGGAGCTGCTTC
*mepA*_RF	AAATGCCGATGTGAAGACTGATATTCCACGCTGGTAAAAAATTCCGGGGATCCGTCGACC
*mepA*_RR	ACAGCGGGGAAACCATAAACAGGCTATTAAACGTTTCCATGTGTAGGCTGGAGCTGCTTC
*yfcA*_RF	GCCTTCCTGCCAGGCGCTACTGGATGAGCACGTGATCTAAATTCCGGGGATCCGTCGACC
*yfcA*_RR	AAATTTCAATCAACTGCTCGTAGTGGTGTGTACTGTTCATGTGTAGGCTGGAGCTGCTTC
*epmC*_RF	CCACGGACAGGAGATCCTCCACTGGTTGGGGATGAACTAAATTCCGGGGATCCGTCGACC
*epmC*_RR	AATTTTGTAGTGCTTTAATGAAGCGTGCCGGACGTTCCGGGTGTAGGCTGGAGCTGCTTC
*yfcL*_RF	CAACTGAAGCCAGCGCCACGTTTTACAATGAGGAAAGAAGATTCCGGGGATCCGTCGACC
*yfcL*_RR	ATAAGCGTCGCATCAGGCATGCTGCTCCAGACGCCGCAACGTGTAGGCTGGAGCTGCTTC
*aspC*_DF	AGAGCAATCTCACGTCTTG

*aspC*_DR	TTCAAAATATTGCAGGGGAC
(*prmB*-*yfcL*)_DF	TGGATATTCCGGAAGATATG
(*prmB*-*yfcL*)_DR	ATCTGCTCTCGCAGTGTTT
*glnALG*_DF	CAGGAGAGTTAAAGTATGTC
*glnALG*_DR	ATTCCAGCATAATGACTCTC
*fabB*_DF	ATCTTAGCGATGTGTGTAAG
*fabB*_DR	TCTTTCAGCTTGCGCATTA
*araFGH*_DF	AACTATGTCAACACAGTCAC
*araFGH*_DR	TACTGCGCGAAAGGAGAAA
*bssS*_DF	AATTCGGTGAATACCCTTAC
*bssS*_DR	ACAACCCAGAAATGGTAAAG
*phoE*_DF	CTGAATATTCGCGCGTTAA
*phoE*_DR	TGAGTTTCACGAGAAGCTG
*cusCFBA*_DF	TTCTGCCATTTTGTGTGGC
*cusCFBA*_DR	TGATCATGCCGCCAATCAT
*ddpXABCDF*_DF	CTACGCTGCACAAATGTTG
*ddpXABCDF*_DR	ATCCAGAAAGCAAAAGCAG
*fdnGHI*_DF	AAAGAGGAAAGCAATGGAC
*fdnGHI*_DR	AAGGGTTTTCATGCCGGAT
*prpBCDE*_DF	GCGGATTAGTTCATGACTT
*prpBCDE*_DR	TTATCCGACCTACGGTTCA
*nanCMS*_DF	AAATACAGCTACCGGTTGC
*nanCMS*_DR	TGAGAACGAACAAGGACAT
*kdpFABC*_DF	GTTAAATCATGGCTTTTGCC
*kdpFABC*_DR	ACAGGCACCGAAGAAAACT
*prmB*_DF	AAACCATTCAGGACATGTTGC
*prmB*_DR	AACACCATCGACGATGCAG
*aroC*_DF	TGTGTTTATGCTCACCAAAG
*aroC*_DR	AACTGCCTATCGATTGTGC
*mepA*_DF	TTGAGATGATCACCAAAGGC
*mepA*_DR	TCAATGCCGGAATGGTGAGT
*yfcA*_DF	AGAACTGCAAAGCTGGTTTG
*yfcA*_DR	TTATACGGTACTTCCGCATC
*epmC*_DF	GTTGAGCAAAGGACAAAAGC
*epmC*_DR	ATTGTAAAACGTGGCGCTG
*yfcL*_DF	ACGCTTCATTAAAGCACTAC
*yfcL*_DR	ATTGTTGAAGGGTAAAGCGG
