# Supplementary material for: Transcriptomic insights into the effects of sublethal exposure to endolysin LNT113 on Escherichia coli
Source: J Microbiol. 2026 Jul 31;64(7):e2605009. doi: 10.71150/jm.2605009 (PMC13714653; doi:10.71150/jm.2605009)
Supplement: Supplementary file 10 [file jm-2605009-Supplementary-Table-S3.pdf]

Table S3. Primers used in plasmid construction

Primer name	Sequence (from 5' to 3')
*fabB*_CF	TAGGGGCCCATGCGCTAAGGCTAAATCC
*fabB*_CR	TAGTCTAGATTAATCTTTCAGCTTGCGCATTACC
promoter_CF	TAGGGGCCCAGTTCCTGCTTGGCCTGCAG
promoter_CR	TAGGAATTCTTAGTATTCCTCCAGGGCATGATC
*aroC*_CF	TAGGAATTCAGGAGGTAATAAATGGCTGGAAACACAATTG
*aroC*_CR	TAGTCTAGATTACCAGCGTGGAATATCA
*mepA*_CF	TAGGAATTCAGGAGGTAATAAATGAATAAAACCGCGATTG
*mepA*_CR	TAGTCTAGATTAGATCACGTGCTCATCCAGTAG
promoter_D1	TGGATTTACACGGTCTGAC
promoter_D2	CTCGCTAAGTGTTGTTTTC
*aroC*_D1	AATTTTGACCTGATCCGGC
*aroC*_D2	AAATCATTGCCCATATGGC
*aroC*_D3	AAACGGATTTTGCTCGACC
*mepA*_D1	TACGCATGACCTGATAATG
*mepA*_D2	GCATATGCATGTACGATTAC
pWSK129_DF	ATTTAGAGCTTGACGGGGAAAG
pWSK129_DR	AGTTAGCTCACTCATTAGGCAC
