# Supplementary material for: Transcriptomic insights into the effects of sublethal exposure to endolysin LNT113 on Escherichia coli
Source: J Microbiol. 2026 Jul 31;64(7):e2605009. doi: 10.71150/jm.2605009 (PMC13714653; doi:10.71150/jm.2605009)
Supplement: Supplementary file 11 [file jm-2605009-Supplementary-Table-S4.pdf]

Table S4. Primer used in RT-qPCR

Primer name	Sequence (from 5' to 3')
*paaJ*_qF	TTTCTCGTCAGGCTGAGATGTTC
*paaJ*_qR	GCATGCTGTCAGTTCCAAATTG
*paaK*_qF	GGAAAAACAAGCGCAACTGATC
*paaK*_qR	TCCAGCTCTTCAATCAGGTTAAGG
*yqeA*_qF	CAGAAATGATTGATGCCGATATG
*yqeA*_qR	CGAGCCACTGTTCATTCTCTTTAC
*astC*_qF	TGAACGAACAGGCGAGTAAGTTC
*astC*_qR	GTGGCGTCGATCAATTTTTTC
*yoeF*_qF	CATGAATCACCCGGTCATTG
*yoeF*_qR	GCTTCCCGTAACCAACTTTTCAC
*dadA*_qF	TGTGATGGCGTTTGGTTCTTAC
*dadA*_qR	CAATTGGAATGGTCAGCGAGTAG
*hcaB*_qF	ATATTCTGGCGGTGGAAGGTAAC
*hcaB*_qR	GCATTGCCGATAAAACAATCC
*aspC*_qF	ACCTCGGGATTGGTGTCTATAAAG
*aspC*_qR	TCATTTTCGAGCAGATACTGTTCAG
*betB*_qF	TGGAACTACCCGATCCAGATTG
*betB*_qR	GGGTAACTTCGCTCGGTTTG
*tyrB*_qF	CGCTTATGGAGCGTTTTAAAGAAG
*tyrB*_qR	GGCTTGCAGTTGTGGAATAATTC
*thrA*_qF	GCCGATATTCTGGAAAGCAATG
*thrA*_qR	CCGCTAATGGTTTTTTCAATCATC
*speE*_qF	TCGCAAACTCAGCCATTACTTC
*speE*_qR	TATCTGTCGCCCATGCAAAAG
*aroC*_qF	AGCCGGATCAGGTCAAAATTC
*aroC*_qR	GCTGGTCAGTGTTTTCGATCAAC
*ltaE*_qF	AACGCGGCGAAGAGTATATTG
*ltaE*_qR	ATGGGTTGCGGTTGAATACTG
*aroD*_qF	TTTCCACCGAGGCTTATATTGC
*aroD*_qR	CATCACCGGTAAATAACTCCAGATC
*cysM*_qF	AAAGGCTATCGCATGAAATTGC
*cysM*_qR	AAGAATCAGTTCCGCACCATAAG
*alaC*_qF	CGCTGGAATTTGCCAAGAAG
*alaC*_qR	GCGAACATGCGTATCACCATAG
*solA*_qF	ATGAACTCTCCCGCCACAAC
*solA*_qR	TGGCGAGAAATGTGGAATCAG
*puuA*_qF	TGGATATTCTGGGCAACGTAGTAG
*puuA*_qR	TAAGGAACCGAGAACAGGAACAC
*pepQ*_qF	CGGGTATCTACTTCATCGAATCG

*pepQ*_qR	ACGGTTTCAGTGCTTCAATTTTC
*cysH*_qF	TCACCGATACGGGTTACTTGTTC
*cysH*_qR	TTTCGGTAGCACGGTACACTTTC
*metA*_qF	TTGCGTGAAGAAAACGTCTTTG
*metA*_qR	AGTTTCAATCTTCTTCGGCATCAG
*ansA*_qF	GAATTATCCGATCAACGAAGTAACG
*ansA*_qR	GAGAGGCAAACGCATCAAAAC
*aroH*_qF	AATCGTAATGCGCACCTATTTTG
*aroH*_qR	AGACCGTGATTTACCCGATAGC
*metC*_qF	CAGCGGTTGCTAATTCCATTC
*metC*_qR	AGAAATCCTGACTCGGTTCATAGG
*avtA*_qF	ACGAATCCAACAGGCAATGTG
*avtA*_qR	GTTATCAATCACCAGCGGAATG
*hisD*_qF	CCGCTCTTCTCAACGGTATTAATG
*hisD*_qR	CATAAAGGATCTCATCGGCAATC
*serA*_qF	AAGGTATCGCTGGAGAAAGACAAG
*serA*_qR	TTGTGAAATTCGATGTTGGTGTAAC
*ybdL*_qF	ACTGCAACCACCGCATTTTC
*ybdL*_qR	ATGCGGAGTGTTGAGGATCAC
*gltB*_qF	TCGAAGCCGACAAAGACTTCTAC
*gltB*_qR	TCCGCAAGATCCAGATAAAAGC
*metL*_qF	AAGAAGCCGATAGCCTCATTAGC
*metL*_qR	CGCATACACTGCGTCGTTAATAC
*trpE*_qF	GGAAAGTGAACAATCACCAAACTG
*trpE*_qR	AAACCGAAAGGGAGCATAAGC
*hisB*_qF	GCAAGGCGTACAGTTTGATGAAG
*hisB*_qR	ACGTTCCACCAGTTTTACTTTCG
*lysA*_qF	AAAGTGGATTCCGTCTCGTTAGG
*lysA*_qR	GATCGATAACATCTGCCGTAAAAAC
*trpB*_qF	CGAAGACGGGCAGATTGAAG
*trpB*_qR	GTGCTGTTAAGATACGCGTGTTG
*fadD*_qF	TTGCTCACACACTGGAAAAAGTG
*fadD*_qR	AATTGACTACCGTGCCTTTTGC
*ucpA*_qF	CGATGACGATCGCGATTTC
*ucpA*_qR	CATCACAATGCGACCATCTTTG
*fabD*_qF	CAATTGTCGAAGAAACGTTTGC
*fabD*_qR	GAGTTTGCCAGGTTTTATTCAGTTC
*fabB*_qF	GGGCAACGTAAAACTGGATACC
*fabB*_qR	TCCATAGAAAGGAATGCATAAATGG
*acpH*_qF	CCTATTACGCTGGATGTCATGTG
*acpH*_qR	CGGGCATAACAGACAAATTCC

*clsC*_qF	AGTCCGGCTTTTTAATCCTTTCTC
*clsC*_qR	TTGTGCATACGGCGATTAAGAC
*psd*_qF	ACGGTATTCTGCGTGAGATGATC
*psd*_qR	TCGTTACGGGCAAACAGATTC
*ispF*_qF	CGAATTGGACACGGTTTTGAC
*ispF*_qR	CCAGCAATCCTTTTTCGTAAGG
*clsB*_qF	ATCGCCAACGCCTACTTCTTC
*clsB*_qR	TCGCCCTGAATGATCAGTTTG
*pgsA*_qF	GACTGACTGGTTCGATGGTTTTC
*pgsA*_qR	GCGATAGCCACGAGAACTTTATC
*glpA*_qF	GCATTAACCAGCATGTGATCAAC
*glpA*_qR	TGGTACCAATCAGCGAAATGG
*glpD*_qF	TGATTCGCATTGGTCTGTTTATG
*glpD*_qR	ACTGAATTTGCGCCAAAACG
*sgcB*_qF	CTTCAACCATGATTGCACACAAG
*sgcB*_qR	GGATCTCATTCAGACAGCATTGG
*yqeF*_qF	CATATGCGGTGGATGAAGTAATTC
*yqeF*_qR	GACCACCTTTAATAGCCGATTGC
*acs*_qF	GGCGCATTATTGATTCCAACTC
*acs*_qR	GCGTCATCAACGTTTTTCTTCAG
*fucO*_qF	TGTACCGATTCTGGCAATTCC
*fucO*_qR	AAACTTGCGCCGTTTCTCTTC
*araB*_qF	GGTTGAAGAAGCGGAAGAGATTAC
*araB*_qR	GAACCATTCGCTGGAATAAATACC
*yihU*_qF	TGCGCTGGTCATTGATATGTC
*yihU*_qR	TCCATCATGCTGAAGCCTTTG
*aldA*_qF	GCTGAAGTCGAAGTGGCTTTTAC
*aldA*_qR	CTGGACGATCGCTTTGAATAATC
*fumA*_qF	CCTTTCATTATCAGGCTCCTTTTC
*fumA*_qR	CAAATTCAGATACGCTAACGTGTTC
*garD*_qF	GCCAACATCGAAATCAGACAAG
*garD*_qR	TTCCTGCTTTCAGGCCATTATC
*crr*_qF	TATTGCTATCAAACCAACGGGTAAC
*crr*_qR	ATAGAGAATGCGTGGTTGGTTTC
*xylA*_qF	TGGAAGCAACCCATAAATTGG
*xylA*_qR	CCTGACGCAAGTCGGTATTTAAC
*fumD*_qF	AAATGTGCAGAGTTGTCGGTAAAG
*fumD*_qR	TGTACGTAATACGCCTGCGATAAC
*ulaA*_qF	GCGTTGAAGACCTCAAATTGC
*ulaA*_qR	AGCAGAATGGCACCAAAGAAG
*gph*_qF	TTGCTGCTGCGGTAGATATGG

*gph*_qR	TCAGAACATCTGCGCCGTTAC
*poxB*_qF	TTCCCTCCAGCGAAATTGG
*poxB*_qR	GAAACCAGCTCGCAATAGTGACTAC
*ybhC*_qF	ATCAACGTCCTTCTGATCAAACC
*ybhC*_qR	TCAGGGATGCAAAATAGTGTTGAG
*scpB*_qF	ACCCGCATGATCCAAAAATTC
*scpB*_qR	CAGATCGGAACTCATGATCATTTC
*manA*_qF	CACAATTCTGAAATCGGTTTTGC
*manA*_qR	CGGCTTGTGGTTAGGATCTTTATAG
*hxpA*_qF	TTTTATGGCGGGCAAATCC
*hxpA*_qR	GCGGTAATACCTTCGGTTTCC
*treB*_qF	GCAAAATAAACCAAACGGATATCG
*treB*_qR	GACAAAGCGTAGGCGAGTAATACAG
*arnB*_qF	GCAAGCTTTTTGCCAGTTGAC
*arnB*_qR	GCCAATTTTCAACGCCATTAG
*ilvM*_qF	TGCAACATCAGGTCAATGTATCG
*ilvM*_qR	TTCATTGAGCAGACGTGGAAAC
*ilvI*_qF	ACGACGAACAATCTGGCAAAG
*ilvI*_qR	CGCAGTCACGGTTTTAGAAATG
*treF*_qF	CCGTCATCGTGATTTTGACTTG
*treF*_qR	TTTTGCGGGTCCGATACATAC
*leuB*_qF	CGCATCGCGTTTGAATCTG
*leuB*_qR	CCGCCATAAAATAGAGGATTGC
*glnA*_qF	TCCCTGCTCATCAGGTGAATG
*glnA*_qR	CGGACTCGTTAATGCCTTTCC
*prpB*_qF	GTTTTGGTTCTTCGGCCTTTAAC
*prpB*_qR	CAACCTGATCTTCAATATGCAATCC
*glcD*_qF	TCTTGTACGAAGAGCGTCTTGATG
*glcD*_qR	ATCGGTATGCAGGATCTCAAGTC
*mepS*_qF	ATATATCTTGCGCGGGATTCC
*mepS*_qR	TGTCTCAGGATGCATATTCTTTGC
*ompW*_qF	AAAGTTAACAGTGGCGGCTTTG
*ompW*_qR	CCTGCACGCATAAAAAATTCG
*nmpC*_qF	TGAATCTCAAGGTTCCTCCAAAG
*nmpC*_qR	TGTCGTATGCTACACCGTAGTTACG
*lpp*_qF	CGCGGTAATCCTGGGTTCTAC
*lpp*_qR	TGCTCAGCTGGTCAACTTTAGC
*nanC*_qF	ATTTTGGCATTCGCTATCGTTAC
*nanC*_qR	TCCCAACGATGAACGTTATCAC
*lolB*_qF	TGGAAGGTTGTTTATGGTGGTTATG
*lolB*_qR	CCATTTTTAACTTGATGCGTTGAC

*mltB*_qF	CAAGCGTCGTTATGTAACATTGC
*mltB*_qR	CGGTGGTCGTATCAGTCTCAGTAG
*lepB*_qF	TGATGCCGACTCTGTTAATTGG
*lepB*_qR	CGATCAGCGTTTTCTGGTAGATAG
*fimI*_qF	CGTAGCGTTGTTTGATGATGAAG
*fimI*_qR	AGCGAAGTAGAGCCTGAATAAAGC
*lsrR*_qF	CGTACGCTAAAAAATGAAAATTGC
*lsrR*_qR	ATTGTCGCATCGTCCTGTTG
*ydcS*_qF	TCCGTATCAACTCACCGAAGAAC
*ydcS*_qR	TCATTTGCACGGTAGTGTCATG
*dksA*_qF	AGCTGATCAAAAAGATCGAGAAGAC
*dksA*_qR	CAGACGGCGAATACCAATTTC
*pdeD*_qF	CGAATGATTGTTTGTACGATTTGC
*pdeD*_qR	GCGAATTGTACTACCCGTTGTTG
*rfaD*_qF	ATGCTACGCTGGCTTATCACAAG
*rfaD*_qR	TTGTCAGATCTGCCTGAGTGAAC
*kdsB*_qF	CATTCGTCAGGTTGCTGATAACC
*kdsB*_qR	TTAAACGCTTCTTCCGCATTG
*lpxH*_qF	TGGTCAGTGCGATGGAAAAAC
*lpxH*_qR	GCTGATTGGCGATAAGTTCATG
*ftsI*_qF	TTCGCGTTCAGCAAGTTTCC
*ftsI*_qR	CAGCCCAAATCGCTTTTACC
*waaH*_qF	AAACCTGGACTGCTCTGGAAATC
*waaH*_qR	TGCGGATAGTTTTCTGCGTAATAC
*mglB*_qF	TGTGATGGCCAGCATGTTATTC
*mglB*_qR	GCGCACTACAGACATAAAGTTATCG
*ytfQ*_qF	GGTAAAAGAAGTGAATGGCAAACC
*ytfQ*_qR	AAGCCTTTCTTACGGTCAATGG
*araF*_qF	AACGCCAAAGGTAAGCCAATG
*araF*_qR	GCCACGTTTCTGCATCTCTTTATAC
*macB*_qF	CAGTTTGTCAGCGGTTTTAACG
*macB*_qR	ATCCCCAGCATGGTCAGTAAAG
*rbsD*_qF	AAAGTACAACGCGTATCGATATGG
*rbsD*_qR	CCTCGACCTGCATTTCATTTG
*argT*_qF	TTATTTCGTCGCTTTCCATTACC
*argT*_qR	CCGCAATCAAACGAGAATCTG
*lolD*_qF	CGGCGATGTGATTTTTAATGG
*lolD*_qR	ACTGATAAATAAAGCCCAGCTTCTG
*tolC*_qF	AGTAACCCGGAATTGCGTAAGTC
*tolC*_qR	AAACCTAGCTGTGGCAGTAATGG
*modF*_qF	CAATGGCGTGGTTTCTTATAACG

*modF*_qR	ATTTGGCCCGACAATTTGC
*hisP*_qF	TCCTCGAAAAACCGAGTGAAG
*hisP*_qR	TTTTATCGGCGACTTTGAGTTG
*potB*_qF	GTTGGCTTGTGTTGTTTGTCTTTC
*potB*_qR	ATCCAGCGTAAAGACCATTTTGAC
*ptsN*_qF	AACCGGTGGACTTACTTTTTGC
*ptsN*_qR	TTTTGTCCGCCAGACGTTTC
*ugpB*_qF	AACAAAGACGCCTTCAAGAAAGC
*ugpB*_qR	TTTCAGTTTCGCGGCATAGTC
*ptsP*_qF	AGGATGCCACGTTACCTTTAATG
*ptsP*_qR	AAACTCGTTTGCCGCTTCTTC
*sapD*_qF	GGCAAGAGTTTGATTGCGAAAG
*sapD*_qR	ATCGATATCATCAAAACGCATACG
*lsrB*_qF	CAAACCGCAGAAGGAATATTAAAAG
*lsrB*_qR	TCAAGTTTTCTGCGGCTTGTG
*metI*_qF	TCTGCGATTGTGAACATTTTCC
*metI*_qR	CAATCGATGTACCGACAATAACG
*oppB*_qF	ATCCGATCATGACACAGTATTTCAG
*oppB*_qR	CAACCAAGTCATTGACCGAATAATC
*livH*_qF	ACACCATGGTTTACGGCATTATC
*livH*_qR	GCGGCGATGATCATAAATGAG
*livJ*_qF	AAACCGCAGCGTATTGCTATC
*livJ*_qR	AGAACACCACGTTTGCATTGC
*potG*_qF	TGGCCTGAAACAGGACAAACTAC
*potG*_qR	TTTGGCGAACTCCTGCATATG
*pstS*_qF	TGCTTTCTCTGTGTTTGCAGAAG
*pstS*_qR	TCTGGTAAGTGTCAGCCCATTTG
*pstC*_qF	GCGCTGATTGTGCTATTGATG
*pstC*_qR	GGAAAGCCAGACCAAATTTCTG
*pstB*_qF	TTTTGGCGTTCGTCTGTTTG
*pstB*_qR	TTGGTTTCGTTCCACAATGC
*phnC*_qF	TGCATGCGGTTGATCTGAAC
*phnC*_qR	AGTGACGTAAAAGGGTGGATTTTC
*dctA*_qF	GTTTGCCGTACTGTTTGGTTTTG
*dctA*_qR	ATGACCTGCGAGAAACTTTCG
*zraP*_qF	CTGGTGACGAAGCGTTATGAATAC
*zraP*_qR	GACTGACGCAAATTCTCCATCTC
*cusC*_qF	CAGCGCCCGGATATTATGG
*cusC*_qR	TATTCCGCTGGTCAGGCTTATAG
*cstA*_qF	ACGGTGGGATGTTAATGGAATC
*cstA*_qR	GGGCTGTTCATGGCAAAATAC

*ddpX*_qF	TAGCCGTAATCTTCCCTGATCTG
*ddpX*_qR	AACGCGCTTGCTGATAAATAGC
*creC*_qF	ACAACATTCTGACGCAAAATGC
*creC*_qR	TCAACAGCAGTCAGAACGACTTC
*fdnI*_qF	TTTGTGCGTTTTGTGCATCAC
*fdnI*_qR	CCACTTTATGCTCATTGCCTTTC
*etk*_qF	CACAACTGAATGGTCAGGATCAG
*etk*_qR	CATACCATTGACGGTGAACTCTTC
*csrA*_qF	CAGGTACGTATTGGCGTAAATGC
*csrA*_qR	TGGACTGCTGGGATTTTTCAG
*glnL*_qF	GCCTGGCACATGAGATTAAAAATC
*glnL*_qR	TTCGAGTAGTGATGGGTCAGGTAAC
*fecA*_qF	GCCTGGTATCTGGATGACAAAATC
*fecA*_qR	TGATGGCGTTGTTCTGGTATG
*rcsF*_qF	AATCAACGCCTCTAAAATGAAAGC
*rcsF*_qR	TAAGCGCAGAACCGATACATACAG
*narL*_qF	CGTCTCTAACCATGAAGAAGATGTG
*narL*_qR	AGCAGATCTTCCGGTTCCATATC
*cpxA*_qF	ATCCGCCCAACGATTTAATG
*cpxA*_qR	TCGGTGGTCACCAATAACAAAC
*rseA*_qF	TGGCTCATAACCCAGAAATGC
*rseA*_qR	CGCGTGAAGAGATATCGAAATG
*ygiM*_qF	CGCCTGATCGGATTAACTTTACTC
*ygiM*_qR	CAGGTATTCAGTTCGTCGGAAAC
*glnD*_qF	GGGCGGAATTAAACGAATGTC
*glnD*_qR	GATCGAATAACAGGCGATTATCG
*dosC*_qF	TTGACGATGTCGAAAGGCTAATAC
*dosC*_qR	AAACCCCATCTCGACAATTTCTAC
*sodA*_qF	GCTGATGGGTGAAGCTATTTCTG
*sodA*_qR	CGGTTCTGGAATTTCAGGTAGTAAG
*degP*_qF	TAGCGATTGGTAACCCGTTTG
*degP*_qR	TTTCGTAGTTTTCGGCATTCAG
*kdpD*_qF	ATCGCGCAGTGGAGTTTTG
*kdpD*_qR	AGGTTTTCTCGCCGCTTTTTAG
*apaH*_qF	CCCTTCTTTCTTGATGCCATGTAC
*apaH*_qR	GGTAAAAGCGTTGGTGATAAAACG
*phoH*_qF	TCTCGTCACTTTGCATTTTCAAC
*phoH*_qR	TGGATAAAATGCCGAAAAACG
*phoB*_qF	GACAGTGCTGTGAATCAACTGAATG
*phoB*_qR	TTGAGGTGTTTGATGAACTGGATAC
*kdpB*_qF	GGATGAGCGGGATCAACATC

*kdpB*_qR	AGTGACCACCGTTAGCCTCAAC
*hokC*_qF	TCGCCCTGATCGTCATCTG
*hokC*_qR	TCTGGCCAGTTCGGATGTG
*fadL*_qF	ATGGAGCTTTGGTCTTGGTTTC
*fadL*_qR	CAGCAGGAGATTGCATAATTTGG
*adeQ*_qF	ATTTTTGCTCACGCTGTTTCG
*adeQ*_qR	AATCCAATTCCGCTGGTGATAC
*exuT*_qF	TCCGGTGCATTGAGCTTTATC
*exuT*_qR	GCGTTCTTCATCGGTCAGATG
*sdaC*_qF	TTCCCGATGACGTTTTTTGC
*sdaC*_qR	TTCTACAACCTCGGTGATGTCTTC
*ppdA*_qF	GCGACCACAGTATCAGCGTTATC
*ppdA*_qR	GTGAACTGCCATGACAGGTATTG
*aqpZ*_qF	CTTCCCGGAATTAGGCATTG
*aqpZ*_qR	CAGAAATATGACCAACAGCAAAGG
*relE*_qF	TCCGTGGTATGCCTGATTGTTAC
*relE*_qR	AAATCACGAAAACGACAACTTTCTC
*nhaA*_qF	TTCACTCGAAATCAACAAAAACATG
*nhaA*_qR	CTTGCATCAGTTCACGTTTAACTTC
*hokD*_qF	CATAGTGACGGCACTGGTAACG
*hokD*_qR	CTCAGGTTCGTAAGCTGTGAAGAC
*yahN*_qF	AAATGCCGAAACACCAACATG
*yahN*_qR	ACAGCGGGCAAAGAAAACG
*cycA*_qF	CAATCTCACAAACCGACATATTCAG
*cycA*_qR	GGCAAGGCTAATCGTTTTGC
*bssS*_qF	TGCACTACCAGACCCCAAATAAG
*bssS*_qR	CGAAATAAATTGTCTGGCAACATC
*hokB*_qF	TCTGCATTACGATTCTGACATTCAC
*hokB*_qR	AGCGCAGCAACCTCCTTATC
*eamB*_qF	CCGAACAATATTCTCGCCCTTAG
*eamB*_qR	CACAATCAAAAATCCCAGACTCATC
*bssR*_qF	CAACTGAGGAAGGCAAAAGGATAC
*bssR*_qR	TTATCGTGCAGTTCGCGATTC
*kdgT*_qF	CCCTGAATTGCGAGAATTTTTC
*kdgT*_qR	TCTGGGCAATCACAGTCAAATC
*crcB*_qF	TTAATCACCACCGGATTTTGTG
*crcB*_qR	TTCAGTAATGCCCAGCCAAAG
*ygbN*_qF	TTACATTGTTATGCATTGCGTTAGC
*ygbN*_qR	AGGAGCAAAGCAACGAATGG
*cirA*_qF	TCTTCCGTGGAACAAAATCTTAAAG
*cirA*_qR	TTTGAGGACATCCTTCAGATTCTG

*aroP*_qF	TGTGCTGATCTTCCACGAGTTAG
*aroP*_qR	TGTTGCAATATACGCAGCTGTTG
*ydhP*_qF	GCATTTTGACCAGCCTGAATC
*ydhP*_qR	CGCTGGCCTGTTTATGTTTTG
*lptD*_qF	GCGAATCTGAACACCAAAGATACC
*lptD*_qR	TTGTTTCATCAGGTCCGCTTTAC
*yccA*_qF	TTATCTCCGCATTCGCCTTTAC
*yccA*_qR	CATAGCGATTACGTCACCCATTC
*tomB*_qF	ACTCACCCAAAAGACATGATATCG
*tomB*_qR	TGATTGCTTTCTTCAAGGTTTGC
*xylE*_qF	AGTGAATCCGCTGCCAACTC
*xylE*_qR	CCGAAGCGGTTACTGCAATAAC
*puuP*_qF	GCCGATGATTAACGTCTTGTTG
*puuP*_qR	AAAATGGCGACGAAGGTTACC
*zitB*_qF	CGCTGGATATCGCTGAACTG
*zitB*_qR	TCTCGCCTACCATCCATACATG
*mdtE*_qF	AGGTGGGCGGTATTATCATTAAAC
*mdtE*_qR	CCTGTAAAGGTGCAGGATCAATC
*blc*_qF	TGGCGTGACCGTAGTAAATAATTTC
*blc*_qR	TTTCCAGTCCACGTTCAAAGC
*mscS*_qF	GCGATCATCATCGTTGGTTTG
*mscS*_qR	CAACAGTGGCATCGATTTTACG
*amtB*_qF	TGATGCTGAAAAACATCGAACTG
*amtB*_qR	CAACTATCAAGCCGACGGTAATG
*ycaD*_qF	CGTTGTCAGCTCATCCTATTTTACC
*ycaD*_qR	TAGCTGCGGTTAAAGCCAATG
*ybhG*_qF	GGGTATCGCAATGAAGAAATCG
*ybhG*_qR	TGCTGGCGGTTATAGAAGTTCTG
*galP*_qF	TGCAGGTAATGCAGCAATTCAC
*galP*_qR	TTGCTCGGTAGTGTTGGTATAACC
*ydeA*_qF	TTGCTCGGTAGTGTTGGTATAACC
*ydeA*_qR	AATAAACACCACAAACAGGCAGATC
*kefF*_qF	ATCCGTATCCGCATCATTCC
*kefF*_qR	GAGTTGATAAAGAGAGCGAATTTCG
*phoE*_qF	TTTTGCCGGGTTGAAATATAAAG
*phoE*_qR	CCAAATTCCGGGAACATATCG
*csgE*_qF	TCATCTATTGGCCATGATTTTTACC
*csgE*_qR	CACTGGGCCTTTCATTAATCG
